# Altered cerebral blood flow and functional connectivity in sickle cell disease

**DOI:** 10.1093/jscdis/yoaf031

**Published:** 2025-09-18

**Authors:** Daniel M Sop, Yue May Zhang, Wally R Smith

**Affiliations:** Division of General Internal Medicine, Department of Internal Medicine & Biomedical Engineering, Virginia Commonwealth University, Richmond, VA 23219-0306, United States; Division of General Internal Medicine, Department of Internal Medicine, Virginia Commonwealth University, Richmond, VA 23219-0306, United States; Division of General Internal Medicine, Department of Internal Medicine, Virginia Commonwealth University, Richmond, VA 23219-0306, United States

**Keywords:** sickle cell disease, cerebral blood flow, resting-state fMRI, functional connectivity, cognitive impairment

## Abstract

**Background:**

Adults with sickle cell disease (SCD) often experience cognitive deficits and chronic pain, but the cerebral mechanisms underlying these symptoms remain unclear. Elevated cerebral blood flow (CBF) is a compensatory response to anemia, yet its impact on brain function and perception is not well understood.

**Objective:**

To examine alterations in cerebral perfusion and resting-state brain function in adults with SCD and their associations with cognition and pain sensitivity.

**Methods:**

Seven adults with SCD and 3 healthy controls underwent arterial spin labeling (ASL) and resting-state functional MRI (rs-fMRI). Metrics included global/regional CBF, resting-state functional connectivity (rsFC), and amplitude of low-frequency fluctuations (ALFF). Participants completed NIH Toolbox fluid cognition tests and the Pain Sensitivity Questionnaire (PSQ).

**Results:**

SCD patients exhibited significantly higher global CBF (72.1 vs. 47.2 mL/100g/min; *P *= .04), reduced cortical zALFF (*P *= .0013), and elevated white-matter zALFF (*P *= .0023). They also showed resting-state network hyperconnectivity, with diminished anti-correlations between the default mode and salience networks. SCD participants scored lower on processing speed (*P *= .02) and reported higher pain sensitivity (PSQ total, *P *= .0040). Higher CBF was associated with slower cognitive performance but not directly with pain sensitivity. Exploratory mediation models suggested that altered brain activity may partially mediate this relationship.

**Conclusions:**

Adults with SCD demonstrate cerebral hyperperfusion, disrupted functional connectivity, and altered spontaneous brain activity, which may contribute to cognitive slowing and heightened pain sensitivity. These findings highlight the need for further research into brain-targeted therapies in SCD.

## INTRODUCTION

Sickle cell disease (SCD) is a hereditary hemoglobinopathy characterized by chronic hemolytic anemia and episodic vaso-occlusive crises.^1^ Beyond its systemic effects, SCD has significant neurological impacts: chronic anemia leads to cerebral hemodynamic adaptations including elevated resting cerebral blood flow (CBF) as a compensatory mechanism to preserve oxygen delivery.^2^ However, this chronic cerebrovascular dilation reduces cerebrovascular reserve and is associated with a “cerebral vascular instability”—a state wherein global hyperperfusion coexists with regional hypoperfusion, predisposing patients to silent cerebral ischemia and infarcts.^3^ Indeed, adults with SCD show higher baseline CBF than healthy controls, yet paradoxically often suffer neurocognitive deficits (particularly in processing speed and executive function) due to diffuse ischemic injury and microinfarcts.^4^ These observations suggest a complex interplay between anemia-driven CBF changes and brain function in SCD.

In parallel, chronic pain is a hallmark of SCD that can lead to central nervous system changes.^5^ Repeated pain crises and ongoing nociceptive input may induce central sensitization and neuroplastic changes in pain-processing circuits.^6^ Patients with SCD frequently report heightened pain sensitivity and neuropathic pain features even at baseline.^7^ Neurologically, chronic pain in non-SCD populations has been linked to alterations in resting-state brain activity and connectivity, such as hyperconnectivity within the salience network (SLN) and between limbic (emotion/pain) regions and executive control regions, along with disruptions in the default mode network’s normal activity.^8^^,^^9^ It is plausible that SCD patients, experiencing lifelong recurrent pain and anemia-related hypoxic stress, exhibit distinctive patterns of resting-state functional connectivity (rsFC) and intrinsic brain activity that underlie both their cognitive deficits and pain modulation.

Resting-state functional MRI provides a window into the brain’s functional organization and baseline neural activity.^10^ Two complementary metrics were of particular interest in this study: (1) rsFC—the temporal correlation of spontaneous low-frequency BOLD signals between brain regions, reflecting communication within and between neural networks^11^ and (2) Amplitude of Low-Frequency Fluctuations (ALFF)—a measure of the magnitude of spontaneous neural activity in the 0.01-0.1 Hz range.^12^ Aberrations in rsFC can reveal network reorganization (eg, loss of normal network segregation or emergence of maladaptive hyperconnections), while ALFF indicates regional neural activity strength or neural “idling” at rest.^13^ Prior work in pediatric SCD has shown reduced ALFF in default-mode network hubs (eg, posterior cingulate cortex [PCC]) alongside increased ALFF in regions like the anterior cingulate (emotion/pain processing),^14^ suggesting that chronic SCD may shift the balance of baseline neural activity. However, comprehensive studies in adults integrating CBF, rsFC, ALFF, and clinical outcomes are scarce.

### Study objective

In this context, we sought to investigate how CBF and resting-state brain function are altered in adults with SCD, and how these alterations relate to cognitive performance and pain experience. Using arterial spin labeling (ASL) MRI and resting-state fMRI, we compared global and regional CBF, rsFC network patterns, and ALFF between adults with SCD and healthy controls. We further examined associations between these neuroimaging metrics and key clinical outcomes—specifically, fluid cognitive abilities (with emphasis on processing speed/executive function) and pain sensitivity (self-reported via standardized questionnaire). We hypothesized that SCD patients would demonstrate elevated CBF, disrupted rsFC (including enhanced connectivity in pain-related networks and reduced inter-network anti-correlations), and altered ALFF relative to controls, and that these brain changes would be linked to worse cognition and heightened pain sensitivity. An exploratory mediation analysis was conducted to test whether alterations in brain functional activity (rsFC/ALFF) mediate the relationship between CBF (reflecting anemia severity) and pain perception in SCD.

## METHODS

### Study design and participants

We performed a cross-sectional observational study of adult SCD patients and demographically similar healthy controls. SCD participants were recruited from an academic medical center’s SCD clinic; inclusion criteria required genotype-confirmed SCD (HbSS, HbSC, or HbSβ^0^ thalassemia), age 18-55, and no acute vaso-occlusive crisis or blood transfusion in the preceding ∼4 weeks. Healthy control (HC) participants were African American adults without SCD or other significant health issues. All participants provided informed consent. Two SCD participants were excluded from fMRI analyses due to structural MRI findings of silent cerebral infarcts. Such lesions can cause signal dropout or distortion in affected regions, which may confound interpretation of functional connectivity results.

### Clinical and neurocognitive assessments

Hematologic and vital parameters were recorded (including hemoglobin concentration and resting heart rate, given their relevance to CBF autoregulation). Cognitive function was evaluated using the NIH Toolbox fluid cognition battery on an iPad, which comprises tests of Processing Speed, Executive Function, and Cognitive Flexibility. In particular, we analyzed the Pattern Comparison Processing Speed Test score (a measure of psychomotor speed and attention), the Flanker Inhibitory Control and Attention Test (an executive function measure of inhibitory control), and the Dimensional Change Card Sort Test (assessing cognitive flexibility/set-shifting).^15^ Pain sensitivity was assessed with the Pain Sensitivity Questionnaire (PSQ), a validated self-report instrument in which individuals rate the intensity of imagined everyday painful scenarios on a 0-10 scale.^16^ The PSQ yields a total score (reflecting overall pain sensitivity) and sub-scores for mild (PSQ-minor) and moderate (PSQ-moderate) pain scenario ratings. Higher PSQ scores indicate greater pain sensitivity.

### MRI acquisition

All participants underwent brain MRI on a 3 Tesla scanner (Philips) with a 32-channel head coil. Anatomical imaging included high-resolution T1-weighted MPRAGE sequences for structural reference and screening of silent infarcts or lesions. CBF imaging was performed using 3D pseudocontinuous ASL (pCASL). In pCASL, a series of label and control images were acquired following labeling of arterial blood in the cervical region (label duration ∼1.5 s, post-label delay ∼2.0 s). Paired label/control images were subtracted to yield perfusion-weighted images, which were then quantified to absolute CBF (ml blood per 100 g tissue per minute) using the Bayesian Inference for ASL (BASIL) toolkit in FSL (Oxford, UK). CBF maps were registered to MNI standard space; global (whole-brain) gray matter CBF and regional CBF values (for regions of interest such as frontal cortex) were extracted for analysis. Additionally, a 2D phase-contrast MRI (Q-flow) sequence at the level of the carotid arteries was acquired in SCD patients to cross-validate whole-brain blood flow (by measuring total flow in internal carotid and vertebral arteries).

### Resting-state fMRI

A gradient-echo echo-planar imaging sequence sensitive to BOLD contrast was used for resting-state fMRI (eyes closed, 6-8 minutes, TR ∼2000 ms, ∼180 volumes). Preprocessing was done in a combined FSL and AFNI pipeline: steps included motion correction, slice timing correction, spatial normalization to MNI space, and spatial smoothing (FWHM ∼5 mm). We applied temporal band-pass filtering (0.01-0.1 Hz) and regression of nuisance signals (motion parameters, ventricular and white matter signals) including ICA-AROMA for artifact removal. Functional connectivity analysis was conducted using a region-of-interest (ROI) approach: we defined ROIs corresponding to key nodes of canonical networks (Default Mode Network, SLN, sensorimotor network, frontoparietal network, etc.), based on an atlas (eg, Automated Anatomical Labeling atlas for cortical regions). Fisher Z-transformed Pearson correlation coefficients were computed between the BOLD time series of each pair of ROIs to generate an rsFC matrix for each subject. We examined network integration/segregation qualitatively via connectivity matrices and circular connectograms (chord diagrams) for the SCD and control groups. Additionally, 2-sample *t*-tests at each ROI–ROI connection were performed to identify connections significantly different between groups. ALFF calculation: The preprocessed resting-state data were also analyzed for ALFF. For each voxel, the time series was fast Fourier transformed to the frequency domain; the power spectrum was integrated over 0.01-0.08 Hz and the square root taken to obtain ALFF. Voxel-wise ALFF maps were then normalized by converting to *z*-scores (zALFF) relative to the whole-brain mean ALFF, to enable comparison of relative regional activity. Mean ALFF in gray matter and white matter was calculated for each subject, and a voxel-wise group comparison (SCD vs HC) was performed to identify regions with significantly different ALFF.

### Statistical analysis

Group comparisons between SCD and controls were conducted using Student’s *t*-tests for continuous variables. Key outcomes compared included: hemoglobin level, global CBF, regional CBF, cognitive test scores, and PSQ scores. Within the SCD group, Pearson correlations were used to explore associations between imaging metrics and clinical outcomes (eg, CBF vs. cognitive scores; CBF vs. PSQ; ALFF or connectivity measures vs. PSQ and cognition). To further explore causal pathways, we constructed a simple mediation model with CBF as the independent variable (X), pain sensitivity (PSQ score) as the dependent outcome (Y), and candidate neural mediators (M) including fluid cognition and brain activity with ALFF. Given the small sample, this mediation was treated as exploratory and was assessed using regression-based methods to estimate the direct effect of CBF on pain and indirect effects through M, without formal inferential testing (insufficient power for a full mediation test). All statistical analyses were conducted in SAS and MATLAB, with *P *< .05 considered significant for primary outcomes. Given the small number of healthy controls, statistical comparisons between groups should be considered exploratory and interpreted with caution. The limited sample size reduces statistical power and increases the possibility that observed differences may reflect sample variability rather than definitive group effects.

## Results

### Participant characteristics and cognitive/pain outcomes

**Figure 1.**
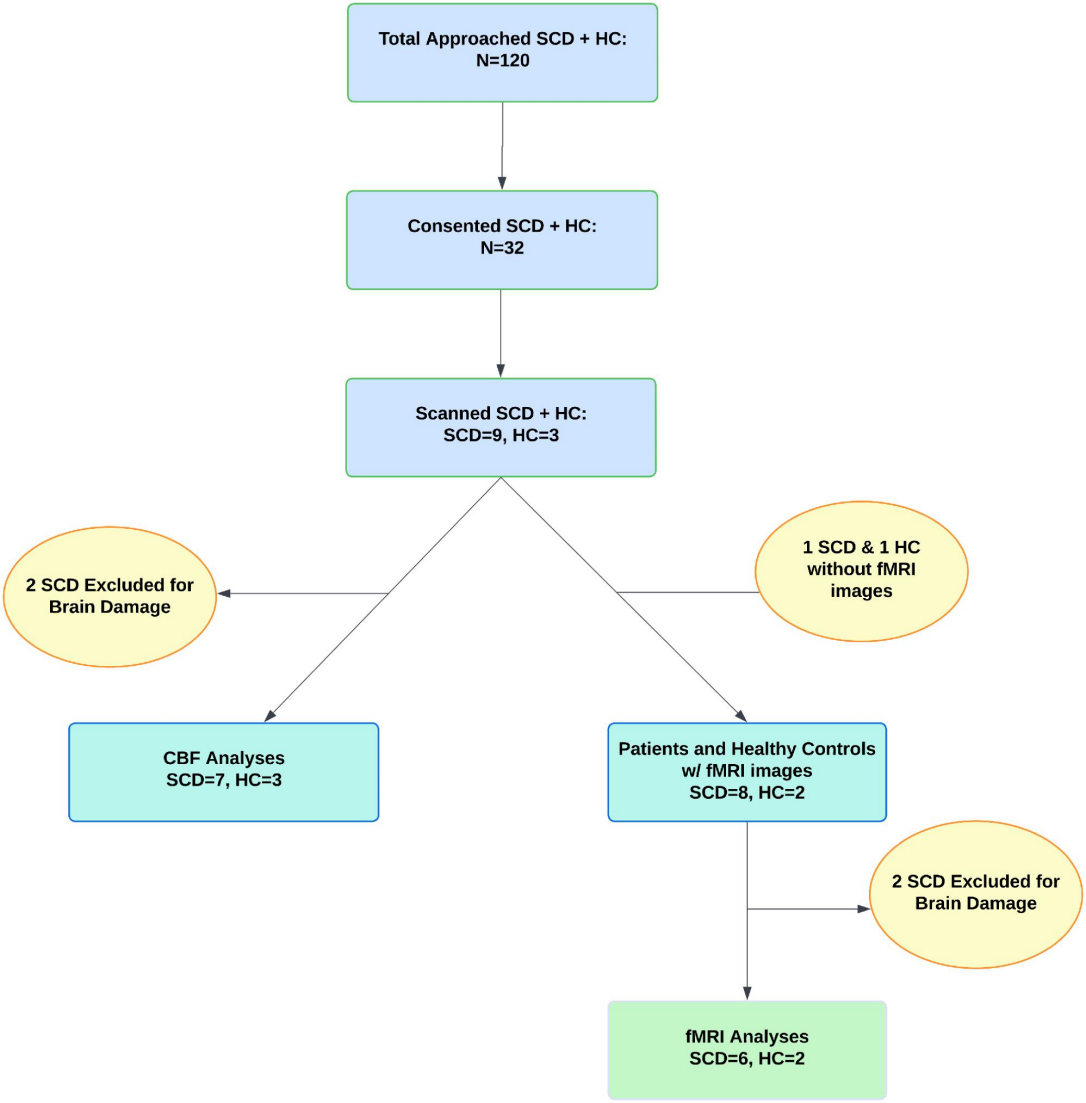
Participant recruitment, exclusions, and final analytic samples. Of the 120 individuals approached (adults with sickle cell disease and healthy controls), 32 consented to participate. MRI scanning was completed in 9 SCD participants and 3 HCs. Two SCD participants were excluded from fMRI analyses due to structural MRI evidence of silent cerebral infarcts, which can cause signal dropout or distortion and confound functional connectivity results. One SCD participant and one HC did not have usable fMRI data and were excluded from those analyses. For cerebral blood flow (CBF) analyses, 7 SCD participants and 3 HCs were included. For resting-state fMRI analyses, 6 SCD participants and 2 HCs remained after exclusions.

All participants were adults (mean age 32 ± 8 years in SCD, thirty in controls; 55% female overall). See Figure 1 and Table 1 for participant recruitment flowchart and patient’s demographics. *Processing speed* was significantly slower in SCD patients (mean Toolbox Pattern Comparison score ∼96.1 vs 123.0 in controls, *P *= .05), indicating a marked deficit in rapid visual scanning and motor response. *Executive function* (Flanker test) and *cognitive flexibility* (Card Sort test) were also lower on average in SCD, though the differences did not reach significance in this small sample (mean Flanker age-adjusted score 79.71 vs 119.33; Card Sort 97.57 vs 115.33, *P *> .1). Consistent with these objective deficits, patients frequently reported subjective “brain fog” or slowed thinking. See Table 2 for fluid cognition score comparisons.

**Table 1.** Patient demographics

Demographic characteristics	Healthy controls (HC)	SCD patients
Sample size (*n*)	3	9
Age in years (mean ± SD)	30.00 ± 7.00	34.13 ± 7.90
Race: Black (*n*, %)	3 (100%)	9 (100%)
Gender: Male (*n*, %)	2 (66.7%)	4 (44.4%)
Gender: Female (*n*, %)	1 (33.3%)	5 (55.6%)
SCD Genotype: SS (*n*, %)	–	6 (66.7%)
SCD Genotype: SC (*n*, %)	–	1 (11.1%)
SCD Genotype: Sβ° (*n*, %)	–	2 (22.2%)

**Table 2.** Fluid cognition in SCD patients vs. healthy controls

	HC (*N* = 3)		SCD (*N* = 7)		*P*-value
	Mean	Std	Mean	Std	
Cognitive flexibility	115.33	22.23	97.57	10.50	.298
Executive function	119.33	28.04	79.71	14.03	.122
Processing speed	123	13.75	96.14	21.04	.054

### Pain sensitivity was markedly elevated in SCD patients

The mean PSQ total score in the SCD group was 5.05 ± 1.63, compared to 2.31 ± 0.50 in healthy controls (*P *= .004). This indicates that, on average, SCD patients rated everyday hypothetical pain scenarios over twice as intensely as controls. PSQ subscales showed a similar pattern: for mild pain scenarios (PSQ-minor), SCD patients scored 4.53 ± 1.48 vs 1.14 ± 0.89 in controls (*P *< .01), and for moderate pain scenarios (PSQ-moderate), 5.57 ± 1.81 vs 3.47 ± 0.36 in controls (*P *= .02). See Table 3 for all PSQ comparisons. These results confirm an enhanced pain sensitivity or lower pain threshold in SCD, even in a steady-state (non-crisis) context. Notably, pain sensitivity did not significantly differ by SCD genotype in this sample—for example, the single HbSC participant actually had a slightly lower PSQ total than the HbSS patients (genotype effect: *F *= 0.17, *P *= .85)—but conclusions on genotype differences are limited by the small numbers per subgroup. In summary, at baseline the SCD cohort demonstrated a combination of cognitive impairment (particularly slower processing speed) and heightened pain perception, reflecting the clinical burden of SCD on the central nervous system. See Table 4 for all relationships.

**Table 3.** Pain Sensitivity Questionnaire: difference between SCD and health controls

	HC (*N* = 3)		SCD (*N* = 7)		*P*-value
	Mean	Std	Mean	Std	
PSQ Total	2.31	0.50	5.05	1.63	.004^a^
PSQ Minor	1.14	0.89	4.53	1.48	.003^a^
PSQ Moderate	3.47	0.36	5.57	1.81	.022^a^

a Indicates statistical significance *P *< .05.

**Table 4.** Impact of genotype of pain sensitivity and fluid cognition

	SB^0^ (mean ± std)	SC (mean ± std)	SS (mean ± std)	*F*-ratio	*P*-value
PSQ total	4.36 ± 1.92	5.92 ± 1.92	5.01 ± 0.86	0.1700	.849
PSQ minor	4.00 ± 1.71	5.57 ± 1.71	4.43 ± 0.76	0.2429	.795
PSQ moderate	4.71 ± 2.15	6.28 ± 2.15	5.60 ± 0.96	0.1354	.877
Cognitive flexibility	90 ± 11.55	106 ± 11.55	97.40 ± 5.16	0.4820	.649
Executive function	86 ± 11.58	101 ± 11.58	74.20 ± 5.18	2.4017	.206
Processing speed	73 ± 21.56	112 ± 21.56	97.60 ± 9.64	0.8581	.490

### Cerebral blood flow: global elevation and regional correlates

**Figure 2.**
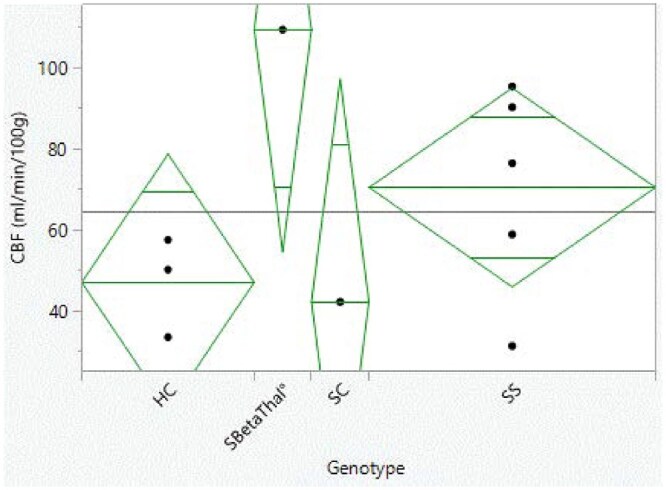
Whole-brain cerebral blood flow in healthy controls and adults with sickle cell disease (SCD), stratified by genotype. Each point represents an individual participant’s mean global gray matter CBF (mL/100 g/min), with group means indicated by horizontal bars. SCD participants show markedly higher CBF than HCs, consistent with anemia-driven hyperperfusion. Green boxes highlight between-group differences meeting the exploratory statistical threshold (*P* < .05) from 2-sample t-tests. Given the small healthy control sample (*n* = 3), these statistical comparisons are preliminary and should be interpreted with caution.

SCD patients showed significantly elevated resting CBF relative to controls (Figure 2). The mean whole-brain CBF in SCD was 72.15 ± 28.9 mL/100g/min, versus 47.23 ± 12.3 mL/100 g/min in healthy controls (*p *= 0.04). Individually, all but one SCD patient had global CBF above the control mean. This hyperperfusion is consistent with chronic anemia-driven vasodilation. Among the SCD group, there was notable heterogeneity: the patient with HbSβ^0^-thalassemia genotype had the highest CBF (∼110 mL/100 g/min), whereas the HbSC patient had a CBF close to the control range. On average, the HbSβ^0^ subject exhibited higher CBF than the HbSS subjects, suggesting a trend toward genotype differences in CBF (more severe anemia genotypes prompting more robust CBF elevation). However, due to the small sample this trend was descriptive only. Figure 2 illustrates CBF values across individuals, highlighting that even the lowest SCD CBF exceeded several control values.

Importantly, the elevated CBF was associated with regional perfusion changes in areas of the brain that subserve cognition. We found that participants with higher whole-brain CBF tended to have higher blood flow in the frontal lobes, but this relationship was not uniform across all frontal regions. In particular, global CBF correlated strongly with perfusion in the middle frontal gyrus (Pearson *r *= 0.76, *P *= .048) and inferior frontal gyrus pars triangularis (*r *= 0.77, *P *= .042). These correlations (illustrated in Table 5) suggest that individuals with the greatest systemic hyperperfusion also had markedly increased blood flow in prefrontal cortical areas associated with working memory, decision-making, and executive function. Other frontal regions (eg, superior frontal gyrus, frontal pole) showed positive correlations (*r* ∼0.67-0.73) that did not reach significance given the small sample size. Previously published,^4^ the relationship between CBF and cognitive performance was probed by correlating CBF with the NIH Toolbox scores: notably, higher CBF was associated with worse processing speed across the combined sample (*r* ≈ –0.8, *P *< .05). In SCD patients, those with the most elevated CBF tended to have the lowest pattern comparison scores (slowest processing), aligning with the hypothesis that extreme hyperemia (reflecting more severe anemia or vasculopathy) may contribute to cognitive slowing. In contrast, CBF was not significantly correlated with PSQ pain sensitivity scores in this small cohort—the regression analysis showed only a nonsignificant trend for higher CBF predicting slightly lower (better) PSQ total (β = –0.058 per mL, *P *= .11) when controlling for genotype. Thus, while anemia-driven CBF elevation was clearly present and linked to cognitive deficits, its direct connection to pain sensitivity is less certain and may involve more complex, indirect pathways.

**Table 5.** Relationship between whole brain blood flow and regional blood flow in the frontal lobe

	CBF	
	Correlation (*R*)	*P*-value
*Frontal Lobe (Cognitive processing centers)*		
Frontal Pole_GM	0.67	.103
Superior Frontal Gyrus_GM	0.71	.073
Middle Frontal Gyrus_GM	0.76	.048^a^
Inferior Frontal Gyrus, pars triangularis_GM	0.77	.042^a^
Inferior Frontal Gyrus, pars opercularis_GM	0.73	.063
Frontal Medial Cortex_GM	0.5	.250
Paracingulate Gyrus_GM	0.73	.060
Cingulate Gyrus, anterior division_GM	0.47	.289
Cingulate Gyrus, posterior division_GM	0.62	.14
Precuneous Cortex_GM	0.56	.190
Frontal Orbital Cortex_GM	0.59	.162
Frontal Operculum Cortex_GM	0.15	.751
Central Opercular Cortex_GM	0.07	.890
Parietal Operculum Cortex_GM	0.68	.080

a Indicates statistical significance *P *< .05.

### Resting-state functional connectivity: network hyperconnectivity in SCD

**Figure 3.**
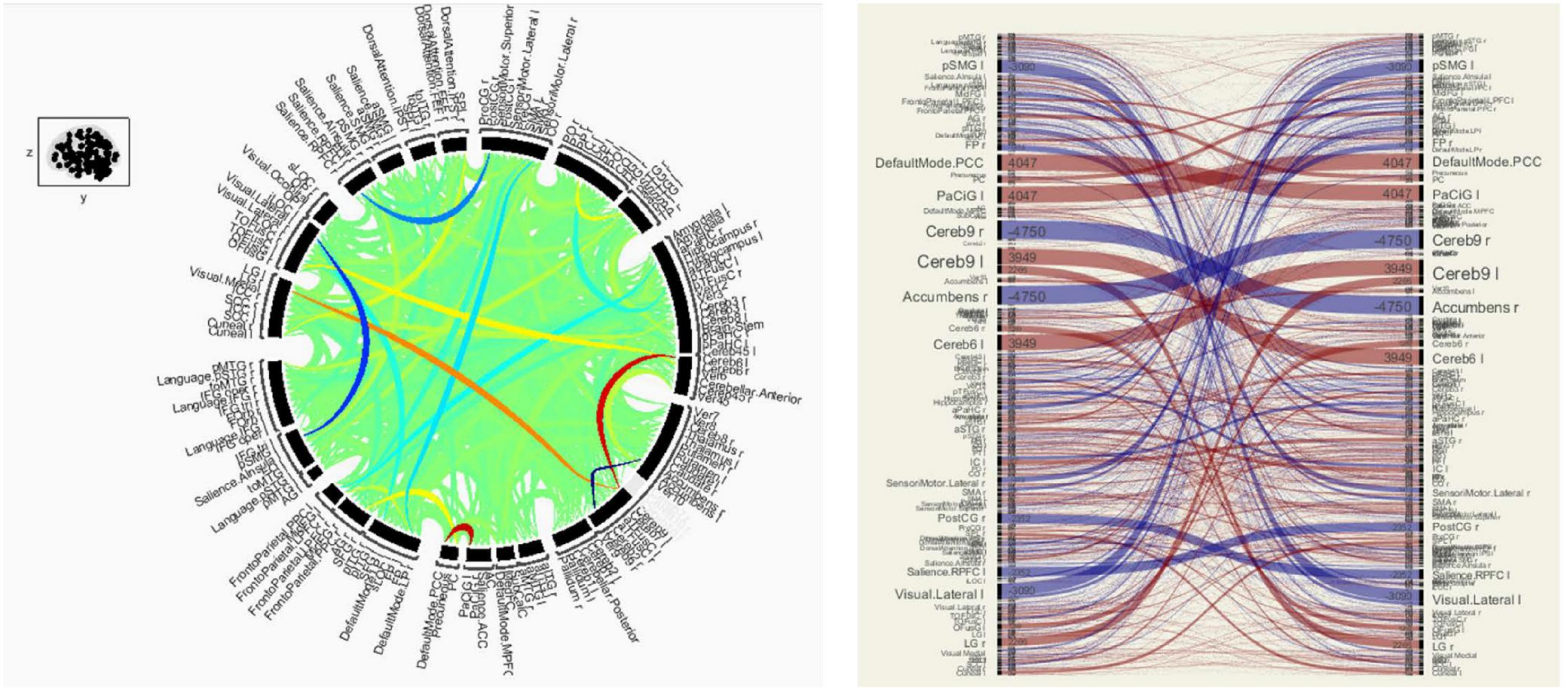
Baseline functional connectivity in healthy controls. Circular and matrix-based visualizations show balanced positive (red/orange) and negative (blue) resting-state correlations. Full names and definitions of all brain nodes are provided in Appendix as Table S1.

Analysis of resting-state fMRI revealed marked alterations in functional connectivity in SCD patients compared to controls. Healthy controls demonstrated the expected balanced pattern of network integration and segregation at rest. Specifically, in controls we observed strong within-network positive connectivity (eg, among regions of the default mode network (DMN), visual network, and sensorimotor network) along with robust between-network anti-correlations—for example, the DMN (a “task-negative” internally directed network) showed negative correlations with task-positive networks like the dorsal attention and SLNs in controls (Figure 3). This is illustrated in the chord diagram for controls where abundant blue lines/arcs indicate these anti-correlations that support dynamic network switching (eg, from resting introspection to focused attention). Key hub regions such as the PCC (a DMN core) and the intraparietal sulcus (a dorsal attention hub) exhibited this balanced reciprocal relationship in controls, reflecting normal functional organization.

**Figure 4.**
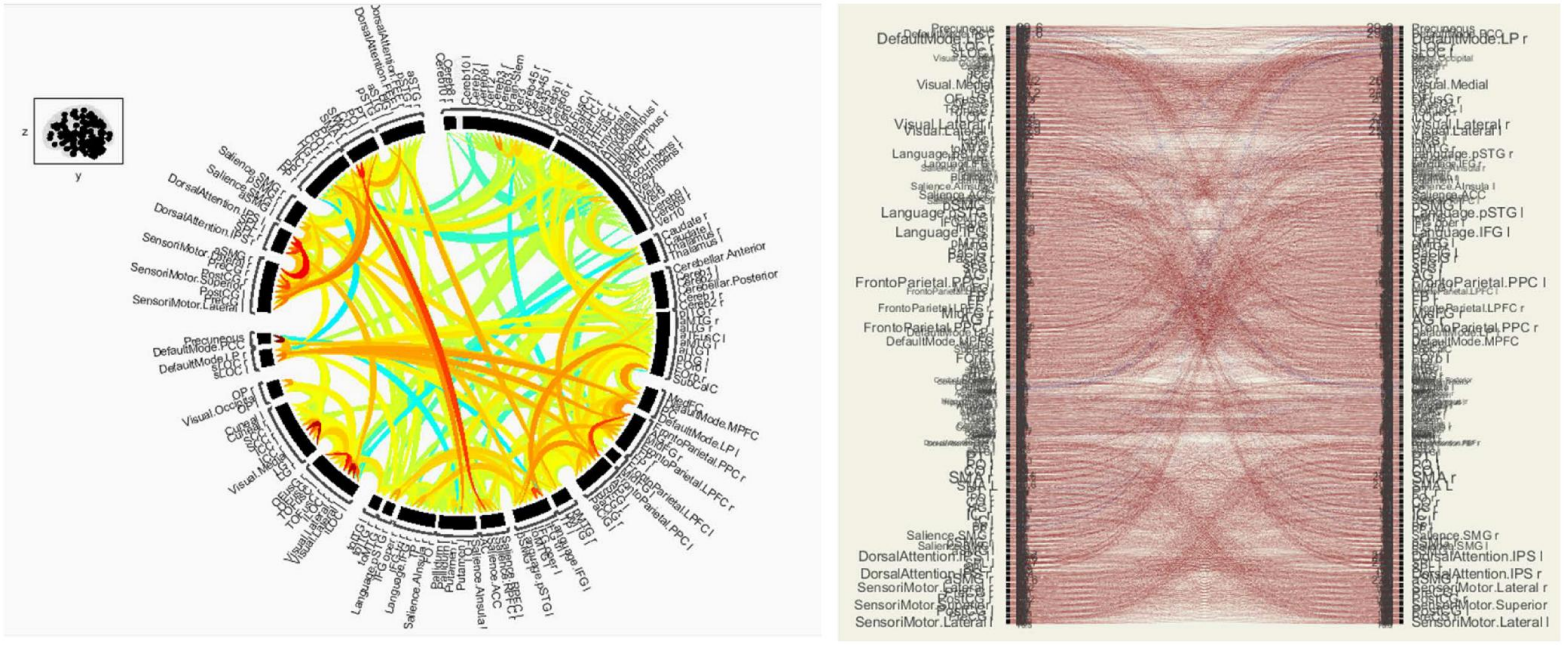
Resting-state functional connectivity in adults with sickle cell disease. Circular and matrix-based visualizations show increased positive correlations (red/orange) and reduced negative correlations (blue) across networks. Full node names are provided in Appendix A as Table S1.

In SCD patients, resting-state connectivity was profoundly different (Figure 4). The same atlas-defined set of nodes was used for both SCD and HC analyses. Differences in visual arrangement between Figures 3 and 4 reflect the default display settings of the connectivity toolbox, which can vary node order to optimize visualization. The apparent differences in node listing do not indicate that different nodes were analyzed between groups. The overall picture was one of diffuse hyperconnectivity: the connectivity matrices and connectograms showed a dominant presence of positive correlations (warm-colored arcs/lines), with a conspicuous reduction in negative correlations between networks. In essence, many brain regions in SCD were more synchronously active than in controls, even between networks that typically oppose each other. For example, anti-correlations between the SLN and the default mode network were markedly blunted in SCD—instead of the clear negative coupling seen in controls, SCD patients showed either weakly negative or even positive coupling between nodes of these networks. Likewise, the normally anti-correlated relationship between the frontal executive network and the DMN was diminished. These findings indicate a loss of normal network segregation in SCD. Many cross-network linkages were hyperconnected, especially those involving regions implicated in pain processing and interoception. For instance, the insula and anterior cingulate cortex (ACC)—key regions in the salience/pain network—were excessively coupled with prefrontal “control” regions and sensorimotor areas in SCD. The insula-precuneus and ACC-dorsolateral prefrontal connectivity were both higher in SCD than controls (qualitatively), suggesting that the boundary between internally oriented and externally oriented networks is blurred. Consistent with this, clusters within the SLN, sensorimotor network, and DMN all showed elevated within-network and between-network synchronization in SCD.

**Figure 5.**
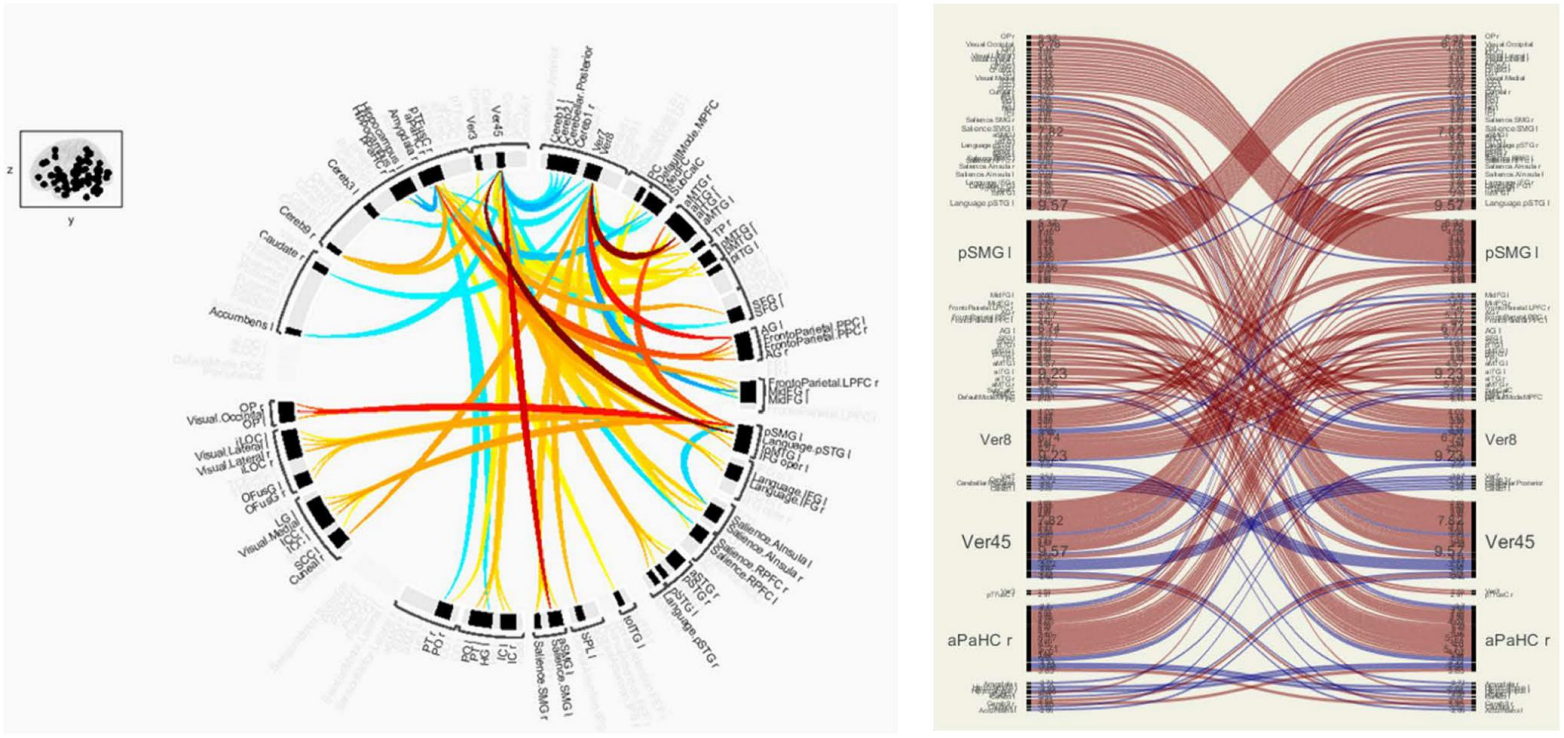
ROI-to-ROI connections (Circular and Matrix view) with significantly greater (red/orange) or reduced (blue) connectivity in SCD compared to healthy controls. Full node names are provided in Appendix A as Table S1.

Statistical ROI-to-ROI group comparisons identified several connections significantly greater in SCD. Notably, we found enhanced cerebellar-cortical connectivity in SCD: for example, the link between the cerebellar vermis (lobules IV/V) and the left superior temporal gyrus (a language network region) was dramatically increased in SCD (*T*(6) = 9.57, *P* < .001). Similarly, connectivity between the cerebellar vermis (lobule VIII) and the left anterior inferior temporal gyrus was elevated (*T*(6) = 9.23, *P* < .001). These indicate that the cerebellum—which contributes to sensorimotor integration and cognitive processing—became more tightly coupled with cortical regions in SCD, possibly reflecting a compensatory reorganization for cortical dysfunction. Another robust difference was greater coupling of the left posterior supramarginal gyrus (pSMG)—part of the perisylvian somatosensory association area and SLN—with multiple regions. The connection between vermis IV/V and left pSMG was highly increased (*T*(6) = 7.82, *P* < .001), as was pSMG coupling to the posterior temporal cortex (*T*(6) = 6.78, *P *= .0005). The right anterior parahippocampal gyrus, a limbic region involved in memory and emotion, also showed increased connectivity to distributed frontal-temporal areas in SCD (*T*(6) ≈ 2.45, *P *= .049). Collectively, these results paint a picture of widespread hyperconnectivity in SCD spanning cerebellar-thalamo-cortical loops, temporoparietal integration areas, and limbic-prefrontal circuits. For visualization, Figure 5 highlights these differences, with red/orange lines indicating connections stronger in SCD and blue lines indicating those weaker than controls. The loss of blue (negative) connections and proliferation of red connections in SCD is readily apparent.

Functionally, this abnormal connectivity suggests that SCD brains may be in a persistent state of high synchronous activity, perhaps due to chronic pain or hypoxia-related neuroplastic changes. The reduced anti-correlations imply an impaired ability to segregate functional networks, which could underlie difficulties in switching between mental states. Indeed, our SCD patients with more blunted network segregation tended to have lower executive function scores (though our sample was too small to yield significant correlations, the direction was consistent). In summary, SCD patients exhibit a resting brain network organization consistent with hyperconnected, less differentiated neural networks, likely reflecting adaptive or maladaptive changes secondary to chronic pain and cerebrovascular stress.

### Intrinsic brain activity (ALFF): decreased cortical and increased white-matter fluctuations

We next examined the amplitude of spontaneous neural activity through ALFF. Striking differences were found in SCD patients compared to controls in both gray and white matter. Global gray-matter ALFF was significantly lower in the SCD group, whereas white-matter ALFF was significantly higher, relative to healthy controls (Table 6). Quantitatively, the SCD patients had a lower mean normalized ALFF (*z*ALFF) in cortical gray matter (by ∼0.5-1 SD units) and a higher mean ALFF in white matter than controls. This was reflected in group comparisons of the mean ALFF within tissue classes: for cortical gray matter, SCD patients had a reduced mean ALFF (normalized) compared to controls (*P *= .002 for mALFF, *P *= .001 for zALFF), indicating diminished spontaneous low-frequency BOLD fluctuations in gray matter. Conversely, in cerebral white matter (where BOLD fluctuations are normally minimal), SCD patients showed a significant elevation in ALFF (*P *= .014 for mALFF, *P *= .002 for zALFF vs. controls). These findings suggest an aberration in resting-state activity: cortical neurons in SCD may be less active or less synchronized at rest, while normally quiescent white-matter regions exhibit abnormally increased BOLD signal fluctuations.

**Table 6.** Regional differences in brain activity (ALFF, mALFF, and zALFF) between SCD patients and controls

Region	ALFF	Control (mean)	SCD (mean)	*P*-value
Whole Brain	ALFF	0.06	0.05	.26
	mALFF	1	1	NA
	zALFF	−3.89E−09	6.00E−11	.16
CSF	ALFF	0.13	0.12	.97
	mALFF	2.15	2.47	.64
	zALFF	1.87	2.55	.53
Grey matter	ALFF	0.06	0.05	.25
	mALFF	0.98	0.97	.002^a^
	zALFF	0.04	0.02	.001^a^
White matter	ALFF	0.04	0.04	.75
	mALFF	0.63	0.75	.014^a^
	zALFF	−0.61	−0.42	.002^a^

a Indicates statistical significance *P *< .05.

Regionally, voxel-wise comparisons (though limited by sample size) pointed to specific foci of ALFF differences consistent with prior reports. SCD patients tended to have lower ALFF in default mode network hubs such as the PCC and medial frontal cortex, as well as in parts of the parietal cortex. In contrast, higher ALFF was observed in SCD in regions like the ACC, insula, and subcortical areas. These patterns align with the aforementioned connectivity findings and literature: for instance, a prior study noted decreased ALFF in PCC and medial orbitofrontal cortex alongside increased ALFF in ACC and cerebellum in pediatric SCD.^9^ Our adult cohort appears to manifest a similar shift of resting activity away from the default-mode regions (perhaps due to damage or disconnection) and towards pain/reward regions like the ACC. Additionally, the increased white-matter ALFF in SCD likely reflects physiological noise from throbbing blood flow or gliosis-related changes; interestingly, periventricular white-matter regions (border-zone areas prone to ischemia) showed some of the greatest ALFF increases in SCD. These ALFF alterations are in line with the notion of chronic ischemia and pain driving changes in both neuronal and neurovascular coupling at rest.

### Associations between neuroimaging metrics, cognition, and pain; mediation analysis

We explored how the above neuroimaging abnormalities related to cognitive and pain measures. As noted, higher global CBF was significantly associated with slower processing speed. However, CBF was not significantly linked to pain sensitivity in this small sample (the trend was toward higher CBF relating to slightly less reported pain sensitivity, but not reaching significance). Interestingly, the rsFC and ALFF measures did not show strong linear correlations with the cognitive or pain scores in this pilot. For example, the overall extent of network hyperconnectivity (eg, average functional connectivity strength) did not correlate in a clear way with PSQ or cognitive composite. Similarly, PSQ scores were not significantly correlated with cognitive scores—SCD patients with high pain sensitivity did not necessarily perform worse or better on cognitive testing (eg, PSQ total vs. processing speed *r *≈ 0.25, and vs. executive function *r *≈ 0.14; both very weak associations). This suggests that, at least in this small group, the variance in cognitive impairment and pain perception may arise from partly distinct mechanisms.

To further dissect the interplay among CBF, brain function, and pain, we conducted an exploratory mediation analysis treating altered brain activity (specifically ALFF) as mediators between CBF and pain sensitivity. The mediation model (though underpowered) yielded insight: it indicated a predominantly direct effect of CBF on pain sensitivity, with only partial mediation by the brain activity metrics. In other words, the pathway from high CBF (anemia severity) to pain outcomes was not largely explained by the measured rs-fMRI abnormalities. One exception was that changes in ALFF in certain regions appeared to partially mediate the CBF–pain relationship. In particular, zALFF in specific frontal-limbic regions showed a small but notable indirect effect: higher CBF was related to lower ALFF in those regions, which in turn was associated with higher pain sensitivity, consistent with a partial mediation. However, the magnitude of this indirect path was limited—the majority of CBF’s total effect on pain (estimated total effect ∼–0.06 units PSQ per unit of CBF) remained as a direct effect not via the brain mediator. These results (summarized in Table 7) underscore that while altered neural activity contributes to pain perception in SCD, it only accounts for a portion of the relationship between physiological disease severity and pain. Other factors (eg, peripheral nociception, inflammation, or unmeasured central factors) likely also play significant roles.

**Table 7.** Mediation analysis of fMRI-derive brain activity in the relationship between cerebral blood flow and pain sensitivity

	NDE (*P*-value)	NIE (*P*-value)	% mediated (*P*-value)
WholeBrain_mean_ALFF	−0.06 (.0003)	0.004 (.58)	−7.84 (.61)
WholeBrain_mean_mALFF	−0.06 (.)	−572e−15 (.)	1.00e−9 (.)
WholeBrain_mean_zALFF			
Csf_mean_ALFF	−0.06 (.0005)	0.001 (.83)	−2.41 (.84)
Csf_mean_mALFF	−0.05 (.002)	−0.003 (.68)	4.66 (.68)
Csf_mean_zALFF	−0.02 (.17)	−0.03 (.07)	56.31 (.04^a^)
GreyMatter_mean_ALFF	−0.06 (.0003)	0.005 (.57)	−8.40 (.59)
GreyMatter_mean_mALFF	−0.10 (.)	0.05 (.)	−80 (.)
GreyMatter_mean_zALFF	−0.07 (<.0001)	0.01 (.30)	−24.82 (.40)
WhiteMatter_mean_ALFF	−0.06 (.001)	−0.00007 (.97)	0.13 (.97)
WhiteMatter_mean_mALFF	−0.09 (<.0001)	0.04 (.03^a^)	−66.32 (.20)
WhiteMatter_mean_zALFF	−0.07 (<.0001)	0.01 (.36)	−25.25 (.46)

a Indicates statistical significance *P *< .05.

In summary, our results demonstrate that adult SCD patients have significant alterations in CBF and brain functional organization, which are linked with their clinical phenotype of cognitive slowing and high pain sensitivity. Specifically: (1) Resting CBF is markedly elevated in SCD and correlates with poorer cognitive performance (slower processing speed). (2) Resting-state networks in SCD are hyperconnected with a loss of normal anti-correlations, suggesting impaired network segregation and possible neural compensatory mechanisms for chronic pain and hypoxia. (3) Baseline neural activity (ALFF) is reduced in cortical regions and increased in normally inactive regions, reflecting disrupted neurovascular homeostasis. (4) SCD patients show both cognitive deficits and heightened pain sensitivity; though these outcomes did not strongly correlate with each other, both appear related to the underlying cerebral pathophysiology. (5) An exploratory mediation analysis indicates that brain functional changes only partly mediate the impact of hemodynamic abnormalities on pain—the direct effect of chronic anemia/CBF on pain suggests other mediators or direct pathways are at play. Additional tables summarizing regression analyses and correlations are provided in the Appendix.

## Discussion

In this study, we integrated multimodal neuroimaging and behavioral assessments to characterize how SCD affects the brain’s blood flow and function. Our findings provide new evidence that adults with SCD experience a substantially altered neurophysiological state at rest, encompassing both perfusion and neural connectivity abnormalities. These brain changes are not merely subclinical curiosities; rather, they have tangible associations with the cognitive and sensory symptoms observed in SCD. Here, we contextualize these results in light of known SCD pathophysiology and prior literature, discuss their implications for understanding SCD-related brain injury and pain, and consider potential clinical implications and directions for future research.

### Elevated CBF as a double-edged sword

We confirmed that SCD patients have significantly higher resting CBF than controls, consistent with chronic compensatory vasodilation due to anemia. This supports prior reports that CBF is inversely related to hemoglobin in SCD, as the brain attempts to maintain oxygen delivery by increasing flow.^17^ While initially adaptive, chronically elevated CBF likely becomes a double-edged sword. On one hand, it indicates the cerebrovascular system is working at maximum capacity to meet metabolic demands. On the other, it erodes the cerebrovascular reserve—the ability to further augment flow when needed is reduced because vessels are already near maximally dilated at baseline. Our finding that higher CBF correlates with slower cognitive processing fits with this interpretation: patients with the greatest hyperemia (often those with more severe anemia) had the poorest cognitive performance. This aligns with a scenario in which chronic hyperperfusion, alongside hemoglobin desaturation, still fails to prevent microischemia in critical brain regions (eg, frontal white matter tracts or cortical areas necessary for processing speed). Over time, high-flow states can cause endothelial shear stress and contribute to cerebral vasculopathy—SCD patients often develop large-vessel stenosis and small-vessel damage in adulthood.^18^ The net effect is a vulnerability to ischemic injury despite increased CBF. Thus, our results reinforce the concept that “more flow” does not equate to “more oxygen” in advanced SCD; beyond a certain point, the coupling of flow and metabolism is perturbed. Clinically, this emphasizes the importance of aggressive anemia management. Interventions like hydroxyurea or transfusions that raise hemoglobin may help reduce the chronic hyperemic drive, potentially improving cognitive outcomes—a hypothesis that could be tested in future longitudinal studies by observing whether normalization of CBF accompanies cognitive benefits.

### Resting-state network dysregulation and cognitive implications

One of the novel contributions of this work is the demonstration of widespread rsFC alterations in SCD adults. We found that SCD brains operate in a more *globally synchronized* manner at rest, with reduced differentiation between networks. This resembles patterns seen in other conditions of brain injury or chronic pain. For example, traumatic brain injury and normal aging have been associated with reduced anti-correlations between default mode and task-positive networks, interpreted as a loss of efficient network switching.^19^ In chronic pain disorders (eg, fibromyalgia), increased connectivity within the SLN and between the insula and prefrontal regions has been reported, reflecting persistent pain-related salience of internal signals.^20^ The SCD connectivity profile—characterized by hyperconnectivity linking pain-related regions (insula, ACC) with cognitive control hubs and sensorimotor areas—suggests that the brain in SCD may be continuously primed in a “pain-ready” state. The attenuation of DMN–SLN anti-correlation in SCD is particularly intriguing. Normally, the SLN (including the insula and ACC) helps toggle the brain between default mode and executive networks in response to salient (eg, painful) stimuli.^21^ In SCD, the insula and ACC were almost uniformly positively coupled with other networks, potentially signifying constant engagement of salience/emotional circuits. This could manifest behaviorally as difficulty in shifting between resting and task states, or as persistent internal focus on discomfort. Though our sample didn’t show a direct correlation between connectivity metrics and cognitive scores, the literature would predict that such network hyperconnectivity might correlate with fatigue, executive dysfunction, or attentional impairments. Indeed, reduced network segregation has been linked to impaired cognitive flexibility, and SCD patients in larger studies have demonstrated deficits in multitasking and attention that might relate to these network-level changes.

It is also worth noting the *cerebellar hyperconnectivity* we observed in SCD. The cerebellum is increasingly recognized for its role in cognitive and affective processing (“cerebellar cognitive affective syndrome”).^22^ Our finding of increased cerebellar–cortical connectivity (eg, vermis to temporal and parietal cortices) in SCD may reflect a compensatory recruitment of cerebellar pathways to support functions compromised by cortical damage. For instance, if frontal–striatal circuits are impaired by chronic ischemia, the cerebellum might partially take over timing and attentional control functions via its connections to cortical association areas. This aligns with the concept of brain network reorganization in response to injury^23^: SCD brains might develop alternate pathways (like greater cerebellar involvement) to maintain function. However, such reorganization might not fully restore normal function and could even be maladaptive in some contexts, potentially explaining why cognitive deficits persist.

### Altered ALFF and neurovascular uncoupling

The ALFF results highlight a fundamental disturbance in the baseline neural activity of SCD patients. Lower cortical ALFF suggests that neuronal activity in low-frequency bands is dampened in many cortical regions. Several factors could contribute to this: chronic ischemia can cause cortical neuronal loss or synaptic depression, leading to reduced spontaneous firing. Also, SCD patients often have chronic pain which might paradoxically “burn out” certain default mode circuits (some studies in chronic pain show reduced DMN activity due to constant engagement by pain).^24^ Meanwhile, the increased ALFF in white matter is unusual—white matter normally has low BOLD signal variance due to fewer synapses and more stable blood flow. Elevated white-matter ALFF in SCD might indicate abnormal fluctuations in cerebral blood volume or oxygenation in these areas. We suspect this finding is related to the phenomenon of hemodynamic instability in watershed white matter regions of SCD brains: as blood flow and oxygen delivery oscillate, the BOLD signal in white matter might fluctuate more than normal, producing a higher ALFF.^25^ Another possibility is that gliosis or inflammation in white matter (from past microinfarcts) could alter neurovascular coupling, leading to measurable BOLD changes.^26^ Regardless, these ALFF changes echo the rsFC findings in suggesting that SCD brains at rest are not in a normal, energy-efficient state. Instead, there is evidence of neurovascular uncoupling, where areas of the brain either under-function (cortex) or show aberrant signals (white matter) relative to metabolic needs. This inefficiency could contribute to symptoms like mental fatigue commonly reported in SCD.

### Pain perception and the brain in SCD

Clinically, SCD is characterized by pain, yet the neurological underpinnings of chronic pain in SCD are not well understood.^27^ Our SCD participants uniformly had elevated PSQ scores, reflecting a generalized hyperalgesic phenotype. The lack of strong correlation between PSQ and cognitive measures implies that pain and cognitive dysfunction might stem from different aspects of SCD pathology. Pain sensitivity in SCD could be driven more by peripheral and spinal mechanisms (eg, repeated vaso-occlusive pain leading to central sensitization in the dorsal horn, or increased small fiber nerve damage). However, our imaging does shed light on central pain processing: the hyperconnectivity of salience and sensorimotor networks in SCD provides a neural correlate for an “always on” pain matrix. Even at rest, the SCD brain may be in a pro-nociceptive state, which could lower the threshold for experiencing pain. Interestingly, our exploratory mediation analysis hinted that some fraction of the relationship between physiological severity (CBF/anemia) and pain might be mediated by brain activity changes—specifically, by altered activity in regions like the ACC or frontal cortex (as captured by zALFF). Although preliminary, this suggests that patients with more extreme anemia and CBF elevation might develop changes in central pain-processing circuits that influence how they perceive pain. For example, chronic hypoxia could damage descending pain modulatory pathways or amplify ACC reactivity, thereby heightening pain sensitivity. The direct effect of CBF on pain (independent of our measured brain metrics) could indicate other mediators such as inflammation or peripheral organ damage (more anemic patients often have more disease complications that cause pain). Future studies with larger cohorts should further examine how neuroimaging markers (like connectivity of the insula or ALFF in pain hubs) relate to quantitative sensory testing and pain diaries in SCD. Understanding this brain–pain relationship could inform better pain management; for instance, if insular hyperactivity is a culprit, treatments targeting central pain processing (like neuromodulation or certain neuromedicines) might be beneficial adjuncts in SCD pain care.

### Limitations

This study is limited by its small sample size and cross-sectional design, which constrain the statistical power and generalizability. The pilot nature means that some findings, especially in the rsFC analysis, were noted at an uncorrected threshold and should be interpreted cautiously until replicated in larger samples. The healthy control group was also small; while the differences observed were large in magnitude, a larger control sample would better establish normative variability. Despite these limitations, the convergence of our results with known pathophysiology (eg, higher CBF with anemia, cognitive slowing with hyperemia, DMN changes with chronic pain) supports their validity. In particular, the very small healthy control group constrains the statistical power of between-group comparisons in Tables 1 and 2, and Figure 2. These findings are presented as preliminary and should be validated in larger, more balanced samples. The small HC sample may also magnify the influence of individual variability on observed network patterns, and some apparent differences in Figures 3 and 4 could reflect this variability rather than systematic group effects.

### Conclusions and future directions

In conclusion, our study provides an in-depth look at the cerebral phenotype of adults with SCD, highlighting a scenario of *compensated yet compromised* brain function. The SCD brain increases blood flow to fight off hypoxia, but in doing so operates on the edge of metabolic insufficiency, leading to subtle brain injuries that manifest as cognitive deficits. Concurrently, the lifelong burden of pain is associated with a brain that is hyperconnected in pain-related networks and altered in baseline activity, which may reinforce the chronic pain cycle. These insights underscore that SCD is as much a disorder of the brain as of the blood, and they invite several future research avenues. Longitudinal studies could determine if these imaging metrics predict the progression of cognitive decline or development of chronic pain in SCD—for instance, could rsFC abnormalities precede neurocognitive symptoms? Interventional studies might explore whether therapies like regular blood transfusions, bone marrow transplant, or novel anti-sickling drugs can normalize CBF and if that yields improvements in cognitive function. Additionally, the role of exercise or cerebrovascular vasodilators (eg, acetazolamide challenges) in teasing out reserve capacity could be informative. From a clinical standpoint, our findings advocate for routine neuropsychological screening in adults with SCD and consideration of brain-directed therapies. Managing SCD should not only aim for preventing strokes, but also mitigating chronic subtle brain changes—through optimizing oxygen delivery, controlling pain, and potentially using cognitive rehabilitation strategies. By doing so, we may improve not just the lifespan but the quality of life and cognitive health of individuals with SCD.

## Supplementary Material

yoaf031_Supplementary_Data

## Data Availability

The data presented in this study are available on request from the corresponding author. The data are not publicly available due to privacy.
