# Supplementary material for: Altered cerebral blood flow and functional connectivity in sickle cell disease
Source: J Sick Cell Dis. 2025 Sep 18;2(1):yoaf031. doi: 10.1093/jscdis/yoaf031 (PMC12476913; doi:10.1093/jscdis/yoaf031)
Supplement: yoaf031_Supplementary_Data [file yoaf031_supplementary_data.docx]

Supplemental Material

Altered Cerebral Blood Flow and Functional Connectivity in Sickle Cell Disease

Daniel M Sop, Ph.D. – [Daniel.sop@vcuhealth.org](mailto:Daniel.sop@vcuhealth.org)

Senior Research Scientist, Division of General Internal Medicine, Department of Internal Medicine & Biomedical Engineering,

Virginia Commonwealth University, Richmond, VA USA.

Box 980306, 730 E. Broad St. Suite 430 Richmond, Virginia, 23219-0306

804-828-8360

ORCID ID: 0000-0003-2076-8319

Yue May Zhang, MS - [Yue.MayZhang@vcuhealth.org](mailto:Yue.MayZhang@vcuhealth.org)

Research Assistant, Division of General Internal Medicine, Department of Internal Medicine

Box 980306, 730 E. Broad St. Suite 430 Richmond, Virginia, 23219-0306

804-828-8360

ORCID iD: 0009-0000-9685-7444

Wally R Smith, MD - [wally.smith@vcuhealth.org](mailto:wally.smith@vcuhealth.org)

Florence Neal Cooper Smith Professor of Sickle Cell Disease,

Vice Chair for Research, Division of General Internal Medicine, Department of Internal Medicine

Virginia Commonwealth University, Richmond, Virginia

Box 980306, 730 E. Broad St. Suite 430 Richmond, Virginia, 23219-0306

804-828-8360

ORCID ID: 0000-0002-4122-5367

#### Corresponding Author contact:

*Daniel M Sop, Ph.D.

[Daniel.sop@vcuhealth.org](mailto:Daniel.sop@vcuhealth.org)

Box 980306, 730 E. Broad St. Suite 430 Richmond, Virginia, 23219-0306

804-828-0810

#### Author Contribution:

Daniel M Sop: Conceptualization, Data curation, Formal analysis, Funding acquisition, Investigation, Methodology, Project administration, Software, Visualization, Writing - original draft.

Yue May Zhang: Formal analysis, Writing - review & editing.

Wally R Smith: Funding acquisition, Resources, Supervision, Writing - review & editing.

ChatGPT was not used.

Conflict of Interest: The authors whose names are listed at the top of this manuscript certify that they have NO affiliations with or involvement in any organization or entity with any financial interest (such as honoraria; educational grants; participation in speakers’ bureaus; membership, employment, consultancies, stock ownership, or other equity interest; and expert testimony or patent-licensing arrangements), or non-financial interest (such as personal or professional relationships, affiliations, knowledge or beliefs) in the subject matter or materials discussed in this manuscript.

#### Acknowledgements

This study was supported (in part) by research funding from the Virginia Commonwealth University Center for Clinical and Translational Research CTSA Grant number (UL1TR002649) from the National Center for Advancing Translational Sciences. Additionally, we acknowledge the administrative, academic and image acquisition support of the following people who worked on various phases of this project: Robert Cadrain, Edward A. Zuniga, and Joel Steinberg, MD.

Funding: This research was funded by Virginia Commonwealth University Center for Clinical and Translational Re-search CTSA and the National Center for Advancing Translational Sciences, grant number UL1TR002649.

Data Availability Statement: The data presented in this study are available on request from the corresponding author. The data are not publicly available due to privacy.

# Appendix

Table S1: Full Brain Node Names and Network Assignments (Figures 3–5)

| Label (as in figures) | Hemisphere | Full anatomical name | Canonical network |
| --- | --- | --- | --- |
| aPaHC r (Parahippocampal Gyrus, anterior division Right)-atlas.AG l (Angular Gyrus Left) |  | A G l ( Angular Gyrus Left) | aPaHC r (Parahippocampal Gyrus, anterior division Right)-atlas Network |
| aPaHC r (Parahippocampal Gyrus, anterior division Right)-atlas.AG r (Angular Gyrus Right) |  | A G r ( Angular Gyrus Right) | aPaHC r (Parahippocampal Gyrus, anterior division Right)-atlas Network |
| Amygdala l | Left | Amygdala | Limbic/Orbitofrontal |
| AG l | Left | Angular Gyrus | Default Mode Network (DMN) |
| AG r | Right | Angular Gyrus | Default Mode Network (DMN) |
| Salience.AInsula l | Left | Anterior Insula | Salience Network (SLN) |
| CO l | Left | Central Opercular Cortex | Salience Network (SLN) |
| aPaHC r (Parahippocampal Gyrus, anterior division Right)-atlas.Cereb9 r (Cerebelum 9 Right) |  | Cereb9 r ( Cerebelum 9 Right) | aPaHC r (Parahippocampal Gyrus, anterior division Right)-atlas Network |
| Ver10 |  | Cerebellar Vermis Lobule X | Cerebellar Network |
| Ver45 |  | Cerebellar Vermis Lobules IV/V | Cerebellar Network |
| Ver45 (Vermis 4 5) -networks.Cerebellar.Posterior (0,-79,-32) |  | Cerebellar. Posterior (0,-79,-32) | Ver45 (Vermis 4 5) -networks Network |
| Cereb6 r | Right | Cerebelum 6 | Cerebellar Network |
| Cereb8 r | Right | Cerebelum 8 | Cerebellar Network |
| Cereb9 r | Right | Cerebelum 9 | Cerebellar Network |
| Cereb2 r | Right | Cerebelum Crus2 | Cerebellar Network |
| pSMG l (Supramarginal Gyrus, posterior division Left)-networks.DefaultMode.MPFC (1,55,-3) |  | Default Mode. M P F C (1,55,-3) | pSMG l (Supramarginal Gyrus, posterior division Left)-networks Network |
| DorsalAttention.FEF l | Left | Frontal Eye Fields | Dorsal Attention Network (DAN) |
| DorsalAttention.FEF r | Right | Frontal Eye Fields | Dorsal Attention Network (DAN) |
| FOrb l | Left | Frontal Orbital Cortex | Limbic/Orbitofrontal |
| aPaHC r (Parahippocampal Gyrus, anterior division Right)-networks.FrontoParietal.LPFC (R) (41,38,30) |  | Fronto Parietal. L P F C ( R) (41,38,30) | aPaHC r (Parahippocampal Gyrus, anterior division Right)-networks Network |
| aPaHC r (Parahippocampal Gyrus, anterior division Right)-networks.FrontoParietal.PPC (L) (-46,-58,49) |  | Fronto Parietal. P P C ( L) (-46,-58,49) | aPaHC r (Parahippocampal Gyrus, anterior division Right)-networks Network |
| aPaHC r (Parahippocampal Gyrus, anterior division Right)-networks.FrontoParietal.PPC (R) (52,-52,45) |  | Fronto Parietal. P P C ( R) (52,-52,45) | aPaHC r (Parahippocampal Gyrus, anterior division Right)-networks Network |
| aPaHC r (Parahippocampal Gyrus, anterior division Right)-atlas.HG l (Heschl's Gyrus Left) |  | H G l ( Heschl's Gyrus Left) | aPaHC r (Parahippocampal Gyrus, anterior division Right)-atlas Network |
| HG r | Right | Heschl's Gyrus | Language/Auditory Network |
| aPaHC r (Parahippocampal Gyrus, anterior division Right)-atlas.Hippocampus l | Left | Hippocampus | aPaHC r (Parahippocampal Gyrus, anterior division Right)-atlas Network |
| aPaHC r (Parahippocampal Gyrus, anterior division Right)-atlas.Hippocampus r | Right | Hippocampus | aPaHC r (Parahippocampal Gyrus, anterior division Right)-atlas Network |
| pSMG l (Supramarginal Gyrus, posterior division Left)-atlas.ICC l (Intracalcarine Cortex Left) |  | I C C l ( Intracalcarine Cortex Left) | pSMG l (Supramarginal Gyrus, posterior division Left)-atlas Network |
| pSMG l (Supramarginal Gyrus, posterior division Left)-atlas.ICC r (Intracalcarine Cortex Right) |  | I C C r ( Intracalcarine Cortex Right) | pSMG l (Supramarginal Gyrus, posterior division Left)-atlas Network |
| aPaHC r (Parahippocampal Gyrus, anterior division Right)-atlas.IFG oper l (Inferior Frontal Gyrus, pars opercularis Left) |  | I F G oper l ( Inferior Frontal Gyrus, pars opercularis Left) | aPaHC r (Parahippocampal Gyrus, anterior division Right)-atlas Network |
| Language.IFG l | Left | Inferior Frontal Gyrus | Language/Auditory Network |
| IFG oper l | Left | Inferior Frontal Gyrus, pars opercularis | Frontoparietal/Executive Control Network (FPN) |
| IFG oper r | Right | Inferior Frontal Gyrus, pars opercularis | Frontoparietal/Executive Control Network (FPN) |
| IFG tri r | Right | Inferior Frontal Gyrus, pars triangularis | Frontoparietal/Executive Control Network (FPN) |
| ICC l | Left | Intracalcarine Cortex |  |
| ICC r | Right | Intracalcarine Cortex |  |
| DorsalAttention.IPS l | Left | Intraparietal Sulcus | Dorsal Attention Network (DAN) |
| DorsalAttention.IPS r | Right | Intraparietal Sulcus | Dorsal Attention Network (DAN) |
| pSMG l (Supramarginal Gyrus, posterior division Left)-atlas.LG l (Lingual Gyrus Left) |  | L G l ( Lingual Gyrus Left) | pSMG l (Supramarginal Gyrus, posterior division Left)-atlas Network |
| FrontoParietal.LPFC l | Left | L P F C | FrontoParietal Network |
| FrontoParietal.LPFC r | Right | L P F C | FrontoParietal Network |
| aPaHC r (Parahippocampal Gyrus, anterior division Right)-networks.Language.IFG (L) (-51,26,2) |  | Language. I F G ( L) (-51,26,2) | aPaHC r (Parahippocampal Gyrus, anterior division Right)-networks Network |
| Ver45 (Vermis 4 5) -networks.Language.IFG (R) (54,28,1) |  | Language. I F G ( R) (54,28,1) | Ver45 (Vermis 4 5) -networks Network |
| aPaHC r (Parahippocampal Gyrus, anterior division Right)-networks.Language.IFG (R) (54,28,1) |  | Language. I F G ( R) (54,28,1) | aPaHC r (Parahippocampal Gyrus, anterior division Right)-networks Network |
| Ver45 (Vermis 4 5) -networks.Language.pSTG (L) (-57,-47,15) |  | Language.p S T G ( L) (-57,-47,15) | Ver45 (Vermis 4 5) -networks Network |
| Ver8 (Vermis 8) -networks.Language.pSTG (L) (-57,-47,15) |  | Language.p S T G ( L) (-57,-47,15) | Ver8 (Vermis 8) -networks Network |
| aPaHC r (Parahippocampal Gyrus, anterior division Right)-networks.Language.pSTG (L) (-57,-47,15) |  | Language.p S T G ( L) (-57,-47,15) | aPaHC r (Parahippocampal Gyrus, anterior division Right)-networks Network |
| Ver45 (Vermis 4 5) -networks.Language.pSTG (R) (59,-42,13) |  | Language.p S T G ( R) (59,-42,13) | Ver45 (Vermis 4 5) -networks Network |
| SensoriMotor.Lateral l | Left | Lateral | Sensorimotor Network (SMN) |
| sLOC l | Left | Lateral Occipital Cortex, superior division |  |
| DefaultMode.LP l | Left | Lateral Parietal Cortex | Default Mode Network (DMN) |
| aPaHC r (Parahippocampal Gyrus, anterior division Right)-atlas.MedFC (Frontal Medial Cortex) |  | Med F C ( Frontal Medial Cortex) | aPaHC r (Parahippocampal Gyrus, anterior division Right)-atlas Network |
| DefaultMode.MPFC |  | Medial Prefrontal Cortex | Default Mode Network (DMN) |
| aPaHC r (Parahippocampal Gyrus, anterior division Right)-atlas.MidFG l (Middle Frontal Gyrus Left) |  | Mid F G l ( Middle Frontal Gyrus Left) | aPaHC r (Parahippocampal Gyrus, anterior division Right)-atlas Network |
| aPaHC r (Parahippocampal Gyrus, anterior division Right)-atlas.MidFG r (Middle Frontal Gyrus Right) |  | Mid F G r ( Middle Frontal Gyrus Right) | aPaHC r (Parahippocampal Gyrus, anterior division Right)-atlas Network |
| MidFG l | Left | Middle Frontal Gyrus |  |
| MidFG r | Right | Middle Frontal Gyrus |  |
| toMTG r | Right | Middle Temporal Gyrus, temporooccipital part |  |
| pSMG l (Supramarginal Gyrus, posterior division Left)-atlas.OFusG l (Occipital Fusiform Gyrus Left) |  | O Fus G l ( Occipital Fusiform Gyrus Left) | pSMG l (Supramarginal Gyrus, posterior division Left)-atlas Network |
| pSMG l (Supramarginal Gyrus, posterior division Left)-atlas.OFusG r (Occipital Fusiform Gyrus Right) |  | O Fus G r ( Occipital Fusiform Gyrus Right) | pSMG l (Supramarginal Gyrus, posterior division Left)-atlas Network |
| pSMG l (Supramarginal Gyrus, posterior division Left)-atlas.OP l (Occipital Pole Left) |  | O P l ( Occipital Pole Left) | pSMG l (Supramarginal Gyrus, posterior division Left)-atlas Network |
| pSMG l (Supramarginal Gyrus, posterior division Left)-atlas.OP r (Occipital Pole Right) |  | O P r ( Occipital Pole Right) | pSMG l (Supramarginal Gyrus, posterior division Left)-atlas Network |
| pSMG l (Supramarginal Gyrus, posterior division Left)-atlas.PC (Cingulate Gyrus, posterior division) |  | P C ( Cingulate Gyrus, posterior division) | pSMG l (Supramarginal Gyrus, posterior division Left)-atlas Network |
| FrontoParietal.PPC l | Left | P P C | FrontoParietal Network |
| FrontoParietal.PPC r | Right | P P C | FrontoParietal Network |
| aPaHC r (Parahippocampal Gyrus, anterior division Right)-atlas.PT r (Planum Temporale Right) |  | P T r ( Planum Temporale Right) | aPaHC r (Parahippocampal Gyrus, anterior division Right)-atlas Network |
| aPaHC r | Right | Parahippocampal Gyrus, anterior division |  |
| Putamen r | Right | Putamen |  |
| Salience.RPFC l | Left | Rostral Prefrontal Cortex | Salience Network (SLN) |
| pSMG l (Supramarginal Gyrus, posterior division Left)-atlas.SCC l (Supracalcarine Cortex Left) |  | S C C l ( Supracalcarine Cortex Left) | pSMG l (Supramarginal Gyrus, posterior division Left)-atlas Network |
| aPaHC r (Parahippocampal Gyrus, anterior division Right)-atlas.SFG r (Superior Frontal Gyrus Right) |  | S F G r ( Superior Frontal Gyrus Right) | aPaHC r (Parahippocampal Gyrus, anterior division Right)-atlas Network |
| Ver45 (Vermis 4 5) -networks.Salience.AInsula (L) (-44,13,1) |  | Salience. A Insula ( L) (-44,13,1) | Ver45 (Vermis 4 5) -networks Network |
| aPaHC r (Parahippocampal Gyrus, anterior division Right)-networks.Salience.AInsula (L) (-44,13,1) |  | Salience. A Insula ( L) (-44,13,1) | aPaHC r (Parahippocampal Gyrus, anterior division Right)-networks Network |
| pSMG l (Supramarginal Gyrus, posterior division Left)-networks.Salience.AInsula (L) (-44,13,1) |  | Salience. A Insula ( L) (-44,13,1) | pSMG l (Supramarginal Gyrus, posterior division Left)-networks Network |
| Ver45 (Vermis 4 5) -networks.Salience.AInsula (R) (47,14,0) |  | Salience. A Insula ( R) (47,14,0) | Ver45 (Vermis 4 5) -networks Network |
| Ver8 (Vermis 8) -networks.Salience.AInsula (R) (47,14,0) |  | Salience. A Insula ( R) (47,14,0) | Ver8 (Vermis 8) -networks Network |
| Ver8 (Vermis 8) -networks.Salience.RPFC (L) (-32,45,27) |  | Salience. R P F C ( L) (-32,45,27) | Ver8 (Vermis 8) -networks Network |
| Ver45 (Vermis 4 5) -networks.Salience.RPFC (R) (32,46,27) |  | Salience. R P F C ( R) (32,46,27) | Ver45 (Vermis 4 5) -networks Network |
| Ver45 (Vermis 4 5) -networks.Salience.SMG (L) (-60,-39,31) |  | Salience. S M G ( L) (-60,-39,31) | Ver45 (Vermis 4 5) -networks Network |
| Ver8 (Vermis 8) -networks.Salience.SMG (L) (-60,-39,31) |  | Salience. S M G ( L) (-60,-39,31) | Ver8 (Vermis 8) -networks Network |
| Ver45 (Vermis 4 5) -networks.Salience.SMG (R) (62,-35,32) |  | Salience. S M G ( R) (62,-35,32) | Ver45 (Vermis 4 5) -networks Network |
| Ver8 (Vermis 8) -networks.Salience.SMG (R) (62,-35,32) |  | Salience. S M G ( R) (62,-35,32) | Ver8 (Vermis 8) -networks Network |
| aPaHC r (Parahippocampal Gyrus, anterior division Right)-atlas.SubCalC (Subcallosal Cortex) |  | Sub Cal C ( Subcallosal Cortex) | aPaHC r (Parahippocampal Gyrus, anterior division Right)-atlas Network |
| SFG l | Left | Superior Frontal Gyrus | Frontoparietal/Executive Control Network (FPN) |
| SPL l | Left | Superior Parietal Lobule | Dorsal Attention Network (DAN) |
| aSTG r | Right | Superior Temporal Gyrus, anterior division |  |
| SCC r | Right | Supracalcarine Cortex |  |
| aSMG l | Left | Supramarginal Gyrus, anterior division |  |
| pSMG l | Left | Supramarginal Gyrus, posterior division |  |
| pSMG r | Right | Supramarginal Gyrus, posterior division |  |
| Thalamus l | Left | Thalamus | Thalamo-cortical |
| Thalamus r | Right | Thalamus | Thalamo-cortical |
| pSMG l (Supramarginal Gyrus, posterior division Left)-atlas.Ver3 (Vermis 3) |  | Ver3 ( Vermis 3) | pSMG l (Supramarginal Gyrus, posterior division Left)-atlas Network |
| Ver6 |  | Vermis 6 | Cerebellar Network |
| Ver9 |  | Vermis 9 | Cerebellar Network |
| pSMG l (Supramarginal Gyrus, posterior division Left)-networks.Visual.Lateral (L) (-37,-79,10) |  | Visual. Lateral ( L) (-37,-79,10) | pSMG l (Supramarginal Gyrus, posterior division Left)-networks Network |
| pSMG l (Supramarginal Gyrus, posterior division Left)-networks.Visual.Lateral (R) (38,-72,13) |  | Visual. Lateral ( R) (38,-72,13) | pSMG l (Supramarginal Gyrus, posterior division Left)-networks Network |
| pSMG l (Supramarginal Gyrus, posterior division Left)-networks.Visual.Medial (2,-79,12) |  | Visual. Medial (2,-79,12) | pSMG l (Supramarginal Gyrus, posterior division Left)-networks Network |
| pSMG l (Supramarginal Gyrus, posterior division Left)-networks.Visual.Occipital (0,-93,-4) |  | Visual. Occipital (0,-93,-4) | pSMG l (Supramarginal Gyrus, posterior division Left)-networks Network |
| aPaHC r (Parahippocampal Gyrus, anterior division Right)-atlas.aITG r (Inferior Temporal Gyrus, anterior division Right) |  | a I T G r ( Inferior Temporal Gyrus, anterior division Right) | aPaHC r (Parahippocampal Gyrus, anterior division Right)-atlas Network |
| pSMG l (Supramarginal Gyrus, posterior division Left)-atlas.aMTG r (Middle Temporal Gyrus, anterior division Right) |  | a M T G r ( Middle Temporal Gyrus, anterior division Right) | pSMG l (Supramarginal Gyrus, posterior division Left)-atlas Network |
| pSMG l (Supramarginal Gyrus, posterior division Left)-atlas.aPaHC r (Parahippocampal Gyrus, anterior division Right) |  | a Pa H C r ( Parahippocampal Gyrus, anterior division Right) | pSMG l (Supramarginal Gyrus, posterior division Left)-atlas Network |
| pSMG l (Supramarginal Gyrus, posterior division Left)-atlas.iLOC l (Lateral Occipital Cortex, inferior division Left) |  | i L O C l ( Lateral Occipital Cortex, inferior division Left) | pSMG l (Supramarginal Gyrus, posterior division Left)-atlas Network |
| pSMG l (Supramarginal Gyrus, posterior division Left)-atlas.iLOC r (Lateral Occipital Cortex, inferior division Right) |  | i L O C r ( Lateral Occipital Cortex, inferior division Right) | pSMG l (Supramarginal Gyrus, posterior division Left)-atlas Network |
| aPaHC r (Parahippocampal Gyrus, anterior division Right)-atlas.pMTG r (Middle Temporal Gyrus, posterior division Right) |  | p M T G r ( Middle Temporal Gyrus, posterior division Right) | aPaHC r (Parahippocampal Gyrus, anterior division Right)-atlas Network |
| pSMG l (Supramarginal Gyrus, posterior division Left)-atlas.pPaHC r (Parahippocampal Gyrus, posterior division Right) |  | p Pa H C r ( Parahippocampal Gyrus, posterior division Right) | pSMG l (Supramarginal Gyrus, posterior division Left)-atlas Network |
| aPaHC r (Parahippocampal Gyrus, anterior division Right)-atlas.pSMG l (Supramarginal Gyrus, posterior division Left) |  | p S M G l ( Supramarginal Gyrus, posterior division Left) | aPaHC r (Parahippocampal Gyrus, anterior division Right)-atlas Network |
| pSMG l (Supramarginal Gyrus, posterior division Left)-atlas.pTFusC r (Temporal Fusiform Cortex, posterior division Right) |  | p T Fus C r ( Temporal Fusiform Cortex, posterior division Right) | pSMG l (Supramarginal Gyrus, posterior division Left)-atlas Network |
| pITG l | Left | posterior Inferior Temporal Gyrus |  |
| pITG r | Right | posterior Inferior Temporal Gyrus |  |
| Language.pSTG l | Left | posterior Superior Temporal Gyrus | Language/Auditory Network |
| pSTG l | Left | posterior Superior Temporal Gyrus |  |
| Language.pSTG r | Right | posterior Superior Temporal Gyrus | Language/Auditory Network |
| pSTG r | Right | posterior Superior Temporal Gyrus |  |
| toITG l | Left | temporo-occipital Inferior Temporal Gyrus |  |
| aPaHC r (Parahippocampal Gyrus, anterior division Right)-atlas.toITG l (Inferior Temporal Gyrus, temporooccipital part Left) |  | to I T G l ( Inferior Temporal Gyrus, temporooccipital part Left) | aPaHC r (Parahippocampal Gyrus, anterior division Right)-atlas Network |

Table S2: Correlation Between Whole-Brain CBF and Regional Blood Flow in the Temporal Lobe: Memory and Auditory Processing Centers

|  | **CBF** | |
| --- | --- | --- |
|  | **Correlation (R)** | ***P*-Value** |
| **Temporal Lobe (Memory/Auditory)** | | |
| Temporal Pole_GM | 0.68 | 0.095 |
| Superior Temporal Gyrus, anterior division_GM | 0.6 | 0.159 |
| Superior Temporal Gyrus, posterior division_GM | 0.71 | 0.073 |
| Middle Temporal Gyrus, anterior division_GM | NA | NA |
| Middle Temporal Gyrus, posterior division_GM | 0.68 | 0.095 |
| Middle Temporal Gyrus, temporooccipital part_GM | NA | NA |
| Inferior Temporal Gyrus, anterior division_GM | 0.54 | 0.215 |
| Inferior Temporal Gyrus, posterior division_GM | 0.73 | 0.062 |
| Inferior Temporal Gyrus, temporooccipital part_GM | NA | NA |
| Temporal Fusiform Cortex, anterior division_GM | 0.34 | 0.451 |
| Temporal Fusiform Cortex, posterior division_GM | 0.4 | 0.373 |
| Temporal Occipital Fusiform Cortex_GM | NA | NA |

Table S3: Correlation Between Whole-Brain CBF and Regional Blood Flow in the Parietal Lobe: Sensory, Touch, and Pain Processing Centers

|  | **CBF** | |
| --- | --- | --- |
|  | **Correlation (R)** | ***P*-Value** |
| **Pariatal Lobe (Sensory/Touch/Pain)** | | |
| Superior Parietal Lobule_GM | 0.04 | 0.925 |
| Supramarginal Gyrus, anterior division_GM | 0.67 | 0.201 |
| Supramarginal Gyrus, posterior division_GM | 0.8 | 0.0312* |
| Angular Gyrus_GM | 0.74 | 0.059 |

* Indicates statistical significance *p* < 0.05.

Table S4: Correlation Between Whole-Brain CBF and Regional Blood Flow in Grey Matter Structures

|  | **CBF** | |
| --- | --- | --- |
|  | **Correlation (R)** | ***P*-Value** |
| **Grey Matter (Cortex)** | | |
| Left Cerebral Cortex_GM | 0.54 | 0.206 |
| Left Putamen_GM | 0.29 | 0.528 |
| Left Pallidum_GM | 0.62 | 0.135 |
| Right Cerebral Cortex_GM | NA | NA |
| Right Putamen_GM | 0.25 | 0.582 |
| Right Pallidum_GM | 0.69 | 0.085 |

Table S5: Connectivity Hubs and Target Regions Implicated in Pain Cognition and Neurocognitive Processing in SCD

| Hub Region | Target Region | *p*-Value | Functional Role |
| --- | --- | --- | --- |
| Vermis 4/5 (Ver45) | ROI 1/164 Ver45 | 0.023* |  |
| Vermis 4/5 (Ver45) | networks.Language.pSTG (L) (-57,-47,15) | < 0.001* |  |
| Vermis 4/5 (Ver45) | networks.Salience.SMG (L) (-60,-39,31) | < 0.001* |  |
| Vermis 4/5 (Ver45) | networks.Language.pSTG (R) (59,-42,13) | < 0.01* |  |
| Vermis 4/5 (Ver45) | atlas.Cereb1 r (Cerebelum Crus1 Right) | < 0.01* |  |
| Vermis 4/5 (Ver45) | atlas.pSTG r (Superior Temporal Gyrus, posterior division Right) | 0.01* | Auditory processing, language comprehension |
| Vermis 4/5 (Ver45) | atlas.Cereb9 r (Cerebelum 9 Right) | 0.011* |  |
| Vermis 4/5 (Ver45) | atlas.PO l (Parietal Operculum Cortex Left) | 0.013* |  |
| Vermis 4/5 (Ver45) | networks.Salience.AInsula (L) (-44,13,1) | 0.017* |  |
| Vermis 4/5 (Ver45) | atlas.IC l (Insular Cortex Left) | 0.021* |  |
| Vermis 4/5 (Ver45) | atlas.PT l (Planum Temporale Left) | 0.022* |  |
| Vermis 4/5 (Ver45) | atlas.IC r (Insular Cortex Right) | 0.025* |  |
| Vermis 4/5 (Ver45) | atlas.Cereb2 l (Cerebelum Crus2 Left) | 0.025* |  |
| Vermis 4/5 (Ver45) | networks.Salience.RPFC (R) (32,46,27) | 0.028* |  |
| Vermis 4/5 (Ver45) | atlas.aSMG l (Supramarginal Gyrus, anterior division Left) | 0.028* | Sensorimotor integration, social cognition, language |
| Vermis 4/5 (Ver45) | atlas.pSTG l (Superior Temporal Gyrus, posterior division Left) | 0.030* | Auditory processing, language comprehension |
| Vermis 4/5 (Ver45) | atlas.pMTG l (Middle Temporal Gyrus, posterior division Left) | 0.033* |  |
| Vermis 4/5 (Ver45) | atlas.PO r (Parietal Operculum Cortex Right) | 0.035* |  |
| Vermis 4/5 (Ver45) | atlas.Accumbens l | 0.037* |  |
| Vermis 4/5 (Ver45) | atlas.toMTG l (Middle Temporal Gyrus, temporooccipital part Left) | 0.041* |  |
| Vermis 4/5 (Ver45) | atlas.Ver7 (Vermis 7) | 0.042* |  |
| Vermis 4/5 (Ver45) | atlas.Cereb3 l (Cerebelum 3 Left) | 0.043* |  |
| Vermis 4/5 (Ver45) | networks.Language.IFG (R) (54,28,1) | 0.044* |  |
| Vermis 4/5 (Ver45) | atlas.SFG l (Superior Frontal Gyrus Left) | 0.044* | Executive function, attention regulation |
| Vermis 4/5 (Ver45) | networks.Salience.SMG (R) (62,-35,32) | 0.045* |  |
| Vermis 4/5 (Ver45) | atlas.aSTG r (Superior Temporal Gyrus, anterior division Right) | 0.045* | Auditory processing, language comprehension |
| Vermis 4/5 (Ver45) | networks.Cerebellar.Posterior (0,-79,-32) | 0.046* |  |
| Vermis 4/5 (Ver45) | networks.Salience.AInsula (R) (47,14,0) | 0.046* |  |
| Vermis 4/5 (Ver45) | atlas.TP r (Temporal Pole Right) | 0.047* |  |
| Vermis 4/5 (Ver45) | atlas.Cereb1 l (Cerebelum Crus1 Left) | 0.047* |  |
| Right Anterior Parahippocampal Gyrus (aPaHC r) | ROI 4/164 aPaHC r | 0.041* |  |
| Right Anterior Parahippocampal Gyrus (aPaHC r) | pSMG l (Supramarginal Gyrus, posterior division Left)-atlas.aPaHC r (Parahippocampal Gyrus, anterior division Right) | 0.041* | Sensorimotor integration, social cognition, language |
| Right Anterior Parahippocampal Gyrus (aPaHC r) | aPaHC r (Parahippocampal Gyrus, anterior division Right)-atlas.AG l (Angular Gyrus Left) | 0.001* | Semantic processing, memory retrieval, default mode network |
| Right Anterior Parahippocampal Gyrus (aPaHC r) | aPaHC r (Parahippocampal Gyrus, anterior division Right)-atlas.AG r (Angular Gyrus Right) | 0.002* | Semantic processing, memory retrieval, default mode network |
| Right Anterior Parahippocampal Gyrus (aPaHC r) | aPaHC r (Parahippocampal Gyrus, anterior division Right)-networks.FrontoParietal.PPC (L) (-46,-58,49) | 0.003* | Memory encoding, emotional regulation |
| Right Anterior Parahippocampal Gyrus (aPaHC r) | aPaHC r (Parahippocampal Gyrus, anterior division Right)-atlas.MidFG r (Middle Frontal Gyrus Right) | 0.005* | Memory encoding, emotional regulation |
| Right Anterior Parahippocampal Gyrus (aPaHC r) | aPaHC r (Parahippocampal Gyrus, anterior division Right)-atlas.IFG oper l (Inferior Frontal Gyrus, pars opercularis Left) | 0.006* | Memory encoding, emotional regulation |
| Right Anterior Parahippocampal Gyrus (aPaHC r) | aPaHC r (Parahippocampal Gyrus, anterior division Right)-atlas.SFG r (Superior Frontal Gyrus Right) | 0.006* | Memory encoding, emotional regulation |
| Right Anterior Parahippocampal Gyrus (aPaHC r) | aPaHC r (Parahippocampal Gyrus, anterior division Right)-atlas.Hippocampus r | 0.007* | Memory encoding, emotional regulation |
| Right Anterior Parahippocampal Gyrus (aPaHC r) | aPaHC r (Parahippocampal Gyrus, anterior division Right)-atlas.Cereb9 r (Cerebelum 9 Right) | 0.008* | Memory encoding, emotional regulation |
| Right Anterior Parahippocampal Gyrus (aPaHC r) | aPaHC r (Parahippocampal Gyrus, anterior division Right)-networks.Language.IFG (R) (54,28,1) | 0.011* | Memory encoding, emotional regulation |
| Right Anterior Parahippocampal Gyrus (aPaHC r) | aPaHC r (Parahippocampal Gyrus, anterior division Right)-atlas.Hippocampus l | 0.012* | Memory encoding, emotional regulation |
| Right Anterior Parahippocampal Gyrus (aPaHC r) | aPaHC r (Parahippocampal Gyrus, anterior division Right)-networks.FrontoParietal.LPFC (R) (41,38,30) | 0.013* | Memory encoding, emotional regulation |
| Right Anterior Parahippocampal Gyrus (aPaHC r) | aPaHC r (Parahippocampal Gyrus, anterior division Right)-atlas.MedFC (Frontal Medial Cortex) | 0.015* | Memory encoding, emotional regulation |
| Right Anterior Parahippocampal Gyrus (aPaHC r) | aPaHC r (Parahippocampal Gyrus, anterior division Right)-networks.FrontoParietal.PPC (R) (52,-52,45) | 0.018* | Memory encoding, emotional regulation |
| Right Anterior Parahippocampal Gyrus (aPaHC r) | aPaHC r (Parahippocampal Gyrus, anterior division Right)-networks.Salience.AInsula (L) (-44,13,1) | 0.021* | Memory encoding, emotional regulation |
| Right Anterior Parahippocampal Gyrus (aPaHC r) | aPaHC r (Parahippocampal Gyrus, anterior division Right)-atlas.pMTG r (Middle Temporal Gyrus, posterior division Right) | 0.022* | Memory encoding, emotional regulation |
| Right Anterior Parahippocampal Gyrus (aPaHC r) | aPaHC r (Parahippocampal Gyrus, anterior division Right)-networks.Language.IFG (L) (-51,26,2) | 0.033* | Memory encoding, emotional regulation |
| Right Anterior Parahippocampal Gyrus (aPaHC r) | aPaHC r (Parahippocampal Gyrus, anterior division Right)-atlas.PT r (Planum Temporale Right) | 0.035* | Memory encoding, emotional regulation |
| Right Anterior Parahippocampal Gyrus (aPaHC r) | aPaHC r (Parahippocampal Gyrus, anterior division Right)-atlas.HG l (Heschl's Gyrus Left) | 0.036* | Memory encoding, emotional regulation |
| Right Anterior Parahippocampal Gyrus (aPaHC r) | aPaHC r (Parahippocampal Gyrus, anterior division Right)-atlas.pSMG l (Supramarginal Gyrus, posterior division Left) | 0.041* | Sensorimotor integration, social cognition, language |
| Right Anterior Parahippocampal Gyrus (aPaHC r) | aPaHC r (Parahippocampal Gyrus, anterior division Right)-atlas.toITG l (Inferior Temporal Gyrus, temporooccipital part Left) | 0.043* | Memory encoding, emotional regulation |
| Right Anterior Parahippocampal Gyrus (aPaHC r) | aPaHC r (Parahippocampal Gyrus, anterior division Right)-atlas.SubCalC (Subcallosal Cortex) | 0.043* | Memory encoding, emotional regulation |
| Right Anterior Parahippocampal Gyrus (aPaHC r) | aPaHC r (Parahippocampal Gyrus, anterior division Right)-atlas.aITG r (Inferior Temporal Gyrus, anterior division Right) | 0.045* | Memory encoding, emotional regulation |
| Right Anterior Parahippocampal Gyrus (aPaHC r) | aPaHC r (Parahippocampal Gyrus, anterior division Right)-atlas.MidFG l (Middle Frontal Gyrus Left) | 0.049* | Memory encoding, emotional regulation |
| Right Anterior Parahippocampal Gyrus (aPaHC r) | aPaHC r (Parahippocampal Gyrus, anterior division Right)-networks.Language.pSTG (L) (-57,-47,15) | 0.049* | Memory encoding, emotional regulation |
| Vermis 8 (Ver8) | atlas.aITG l (Inferior Temporal Gyrus, anterior division Left) | < 0.001* | Postural control, visual-spatial processing |
| Vermis 8 (Ver8) | atlas.AG l (Angular Gyrus Left) | < 0.001* | Postural control, visual-spatial processing |
| Vermis 8 (Ver8) | atlas.aMTG l (Middle Temporal Gyrus, anterior division Left) | 0.003* | Postural control, visual-spatial processing |
| Vermis 8 (Ver8) | atlas.Cuneal r (Cuneal Cortex Right) | 0.006* | Postural control, visual-spatial processing |
| Vermis 8 (Ver8) | atlas.MidFG r (Middle Frontal Gyrus Right) | 0.007* | Postural control, visual-spatial processing |
| Vermis 8 (Ver8) | atlas.SPL l (Superior Parietal Lobule Left) | 0.011* | Postural control, visual-spatial processing |
| Vermis 8 (Ver8) | networks.Salience.SMG (L) (-60,-39,31) | 0.015* | Postural control, visual-spatial processing |
| Vermis 8 (Ver8) | networks.Language.pSTG (L) (-57,-47,15) | 0.017* | Postural control, visual-spatial processing |
| Vermis 8 (Ver8) | networks.Salience.SMG (R) (62,-35,32) | 0.017* | Postural control, visual-spatial processing |
| Vermis 8 (Ver8) | networks.Salience.AInsula (R) (47,14,0) | 0.017* | Postural control, visual-spatial processing |
| Vermis 8 (Ver8) | atlas.pMTG l (Middle Temporal Gyrus, posterior division Left) | 0.022* | Postural control, visual-spatial processing |
| Vermis 8 (Ver8) | atlas.MidFG l (Middle Frontal Gyrus Left) | 0.024* | Postural control, visual-spatial processing |
| Vermis 8 (Ver8) | atlas.TP r (Temporal Pole Right) | 0.028* | Postural control, visual-spatial processing |
| Vermis 8 (Ver8) | atlas.Amygdala r | 0.034* | Postural control, visual-spatial processing |
| Vermis 8 (Ver8) | atlas.pITG l (Inferior Temporal Gyrus, posterior division Left) | 0.035* | Postural control, visual-spatial processing |
| Vermis 8 (Ver8) | networks.Salience.RPFC (L) (-32,45,27) | 0.037* | Postural control, visual-spatial processing |
| Vermis 8 (Ver8) | atlas.aITG r (Inferior Temporal Gyrus, anterior division Right) | 0.039* | Postural control, visual-spatial processing |
| Vermis 8 (Ver8) | atlas.Caudate r | 0.045* | Postural control, visual-spatial processing |
| Vermis 8 (Ver8) | atlas.SubCalC (Subcallosal Cortex) | 0.046* | Postural control, visual-spatial processing |
| Left Posterior Supramarginal Gyrus (pSMG l) | pSMG l (Supramarginal Gyrus, posterior division Left)-networks.Visual.Occipital (0,-93,-4) | < 0.001* | Sensorimotor integration, social cognition, language |
| Left Posterior Supramarginal Gyrus (pSMG l) | pSMG l (Supramarginal Gyrus, posterior division Left)-atlas.aMTG r (Middle Temporal Gyrus, anterior division Right) | 0.001* | Sensorimotor integration, social cognition, language |
| Left Posterior Supramarginal Gyrus (pSMG l) | pSMG l (Supramarginal Gyrus, posterior division Left)-atlas.OP r (Occipital Pole Right) | 0.001* | Sensorimotor integration, social cognition, language |
| Left Posterior Supramarginal Gyrus (pSMG l) | pSMG l (Supramarginal Gyrus, posterior division Left)-atlas.OP l (Occipital Pole Left) | 0.005* | Sensorimotor integration, social cognition, language |
| Left Posterior Supramarginal Gyrus (pSMG l) | pSMG l (Supramarginal Gyrus, posterior division Left)-atlas.ICC l (Intracalcarine Cortex Left) | 0.006* | Sensorimotor integration, social cognition, language |
| Left Posterior Supramarginal Gyrus (pSMG l) | pSMG l (Supramarginal Gyrus, posterior division Left)-atlas.iLOC r (Lateral Occipital Cortex, inferior division Right) | 0.007* | Sensorimotor integration, social cognition, language |
| Left Posterior Supramarginal Gyrus (pSMG l) | pSMG l (Supramarginal Gyrus, posterior division Left)-networks.Visual.Medial (2,-79,12) | 0.007* | Sensorimotor integration, social cognition, language |
| Left Posterior Supramarginal Gyrus (pSMG l) | pSMG l (Supramarginal Gyrus, posterior division Left)-networks.Visual.Lateral (R) (38,-72,13) | 0.013* | Sensorimotor integration, social cognition, language |
| Left Posterior Supramarginal Gyrus (pSMG l) | pSMG l (Supramarginal Gyrus, posterior division Left)-atlas.OFusG r (Occipital Fusiform Gyrus Right) | 0.014* | Sensorimotor integration, social cognition, language |
| Left Posterior Supramarginal Gyrus (pSMG l) | pSMG l (Supramarginal Gyrus, posterior division Left)-networks.Visual.Lateral (L) (-37,-79,10) | 0.015* | Sensorimotor integration, social cognition, language |
| Left Posterior Supramarginal Gyrus (pSMG l) | pSMG l (Supramarginal Gyrus, posterior division Left)-atlas.OFusG l (Occipital Fusiform Gyrus Left) | 0.017* | Sensorimotor integration, social cognition, language |
| Left Posterior Supramarginal Gyrus (pSMG l) | pSMG l (Supramarginal Gyrus, posterior division Left)-atlas.LG l (Lingual Gyrus Left) | 0.020* | Sensorimotor integration, social cognition, language |
| Left Posterior Supramarginal Gyrus (pSMG l) | pSMG l (Supramarginal Gyrus, posterior division Left)-networks.Salience.AInsula (L) (-44,13,1) | 0.022* | Sensorimotor integration, social cognition, language |
| Left Posterior Supramarginal Gyrus (pSMG l) | pSMG l (Supramarginal Gyrus, posterior division Left)-atlas.pTFusC r (Temporal Fusiform Cortex, posterior division Right) | 0.024* | Sensorimotor integration, social cognition, language |
| Left Posterior Supramarginal Gyrus (pSMG l) | pSMG l (Supramarginal Gyrus, posterior division Left)-atlas.ICC r (Intracalcarine Cortex Right) | 0.027* | Sensorimotor integration, social cognition, language |
| Left Posterior Supramarginal Gyrus (pSMG l) | pSMG l (Supramarginal Gyrus, posterior division Left)-networks.DefaultMode.MPFC (1,55,-3) | 0.035* | Sensorimotor integration, social cognition, language |
| Left Posterior Supramarginal Gyrus (pSMG l) | pSMG l (Supramarginal Gyrus, posterior division Left)-atlas.iLOC l (Lateral Occipital Cortex, inferior division Left) | 0.039* | Sensorimotor integration, social cognition, language |
| Left Posterior Supramarginal Gyrus (pSMG l) | pSMG l (Supramarginal Gyrus, posterior division Left)-atlas.pPaHC r (Parahippocampal Gyrus, posterior division Right) | 0.043* | Sensorimotor integration, social cognition, language |
| Left Posterior Supramarginal Gyrus (pSMG l) | pSMG l (Supramarginal Gyrus, posterior division Left)-atlas.Ver3 (Vermis 3) | 0.044* | Sensorimotor integration, social cognition, language |
| Left Posterior Supramarginal Gyrus (pSMG l) | pSMG l (Supramarginal Gyrus, posterior division Left)-atlas.PC (Cingulate Gyrus, posterior division) | 0.048* | Sensorimotor integration, social cognition, language |
| Left Posterior Supramarginal Gyrus (pSMG l) | pSMG l (Supramarginal Gyrus, posterior division Left)-atlas.SCC l (Supracalcarine Cortex Left) | 0.049* | Sensorimotor integration, social cognition, language |

* Indicates statistical significance *p* < 0.05.

Table S6: Correlation Between Cerebral Blood Flow and Brain Activity Metrics (ALFF, mALFF, zALFF) Across Brain Regions

| **Region** | **Metric** | **Correlation (R)** | ***p*-value** |
| --- | --- | --- | --- |
| **Whole Brain** | ALFF | -0.19 | 0.71 |
|  | mALFF | 0 | 1 |
|  | zALFF | -0.01 | 0.98 |
| **CSF** | ALFF | -0.34 | 0.51 |
|  | mALFF | -0.54 | 0.27 |
|  | zALFF | -0.76 | 0.08 |
| **Grey Matter** | ALFF | -0.17 | 0.75 |
|  | mALFF | 0.7 | 0.12 |
|  | zALFF | 0.67 | 0.15 |
| **White Matter** | ALFF | -0.5 | 0.31 |
|  | mALFF | -0.6 | 0.21 |
|  | zALFF | -0.53 | 0.28 |

Table S7: Regression Analysis of CBF Effects on fMRI Activity Metrics (ALFF, mALFF, zALFF) Across Brain Regions

| Whole Brain | | | | | | CSF | | | | | |
| --- | --- | --- | --- | --- | --- | --- | --- | --- | --- | --- | --- |
|  | **Term** | **Estimate** | **Std Error** | **t Ratio** | **Prob>\|t\|** |  | **Term** | **Estimate** | **Std Error** | **t Ratio** | **Prob>\|t\|** |
| ALFF | Intercept | 0.046161 | 0.011569 | 3.99 | 0.057 | ALFF | Intercept | 0.1164694 | 0.056549 | 2.06 | 0.175 |
|  | CBF | 5.55E-05 | 0.000148 | 0.37 | 0.744 |  | CBF | 9.04E-05 | 0.000724 | 0.12 | 0.912 |
|  | Genotype[SBetaThal°] | -0.006674 | 0.007899 | -0.84 | 0.487 |  | Genotype[SBetaThal°] | -0.030955 | 0.038611 | -0.8 | 0.506 |
|  | Genotype[SC] | 0.00735 | 0.007458 | 0.99 | 0.428 |  | Genotype[SC] | 0.0279768 | 0.036452 | 0.77 | 0.523 |
|  | **Term** | **Estimate** | **Std Error** | **t Ratio** | **Prob>\|t\|** |  | **Term** | **Estimate** | **Std Error** | **t Ratio** | **Prob>\|t\|** |
| mALFF | Intercept | 1 | 0 | . | . | mALFF | Intercept | 2.557401 | 0.525985 | 4.86 | 0.039* |
|  | CBF | 0 | 0 | . | . |  | CBF | -0.00181 | 0.006735 | -0.27 | 0.813 |
|  | Genotype[SBetaThal°] | 0 | 0 | . | . |  | Genotype[SBetaThal°] | -0.264714 | 0.359131 | -0.74 | 0.537 |
|  | Genotype[SC] | 0 | 0 | . | . |  | Genotype[SC] | 0.1737867 | 0.339055 | 0.51 | 0.659 |
|  | **Term** | **Estimate** | **Std Error** | **t Ratio** | **Prob>\|t\|** |  | **Term** | **Estimate** | **Std Error** | **t Ratio** | **Prob>\|t\|** |
| zALFF | Intercept | -7.89E-10 | 3.87E-09 | -0.2 | 0.857 | ZALFF | Intercept | 3.0874097 | 0.518352 | 5.96 | 0.027* |
|  | CBF | 5.68E-12 | 4.95E-11 | 0.11 | 0.919 |  | CBF | -0.009728 | 0.006637 | -1.47 | 0.280 |
|  | Genotype[SBetaThal°] | -7.41E-10 | 2.64E-09 | -0.28 | 0.805 |  | Genotype[SBetaThal°] | -0.268055 | 0.353919 | -0.76 | 0.527 |
|  | Genotype[SC] | -1.45E-10 | 2.49E-09 | -0.06 | 0.959 |  | Genotype[SC] | -0.047489 | 0.334134 | -0.14 | 0.9 |
| Grey Matter | | | | | | White matter | | | | | |
|  | **Term** | **Estimate** | **Std Error** | **t Ratio** | **Prob>\|t\|** |  | **Term** | **Estimate** | **Std Error** | **t Ratio** | **Prob>\|t\|** |
| ALFF | Intercept | 0.044723 | 0.011233 | 3.98 | 0.057 | ALFF | Intercept | 0.0367285 | 0.00759 | 4.84 | 0.040* |
|  | CBF | 5.64E-05 | 0.000144 | 0.39 | 0.732 |  | CBF | -2.17E-06 | 9.72E-05 | -0.02 | 0.984 |
|  | Genotype[SBetaThal°] | -0.006271 | 0.00767 | -0.82 | 0.499 |  | Genotype[SBetaThal°] | -0.005991 | 0.005183 | -1.16 | 0.367 |
|  | Genotype[SC] | 0.007102 | 0.007241 | 0.98 | 0.430 |  | Genotype[SC] | 0.0042118 | 0.004893 | 0.86 | 0.480 |
|  | **Term** | **Estimate** | **Std Error** | **t Ratio** | **Prob>\|t\|** |  | **Term** | **Estimate** | **Std Error** | **t Ratio** | **Prob>\|t\|** |
| mALFF | Intercept | 0.968936 | 0.00278 | 348.49 | <.0001* | mALFF | Intercept | 0.7939772 | 0.057451 | 13.82 | 0.005* |
|  | CBF | 5.53E-05 | 3.56E-05 | 1.55 | 0.260 |  | CBF | -0.000911 | 0.000736 | -1.24 | 0.341 |
|  | Genotype[SBetaThal°] | 0.004533 | 0.001898 | 2.39 | 0.139 |  | Genotype[SBetaThal°] | -0.024805 | 0.039226 | -0.63 | 0.591 |
|  | Genotype[SC] | -0.000743 | 0.001792 | -0.41 | 0.718 |  | Genotype[SC] | -0.024129 | 0.037033 | -0.65 | 0.581 |
|  | **Term** | **Estimate** | **Std Error** | **t Ratio** | **Prob>\|t\|** |  | **Term** | **Estimate** | **Std Error** | **t Ratio** | **Prob>\|t\|** |
| zALFF | Intercept | 0.020166 | 0.00772 | 2.61 | 0.120 | zALFF | Intercept | -0.384583 | 0.130123 | -2.96 | 0.097 |
|  | CBF | 6.62E-05 | 9.89E-05 | 0.67 | 0.572 |  | CBF | -0.000902 | 0.001666 | -0.54 | 0.642 |
|  | Genotype[SBetaThal°] | 0.007359 | 0.005271 | 1.4 | 0.297 |  | Genotype[SBetaThal°] | -0.046234 | 0.088845 | -0.52 | 0.654 |
|  | Genotype[SC] | -0.002781 | 0.004977 | -0.56 | 0.632 |  | Genotype[SC] | -0.003971 | 0.083879 | -0.05 | 0.966 |

* Indicates statistical significance *p* < 0.05.

Table S8: Regression Analysis of Brain Activity and Genotype Effects on Pain Sensitivity

| **Whole Brain** | | | | | **CSF** | | | | |
| --- | --- | --- | --- | --- | --- | --- | --- | --- | --- |
| **Term** | **Estimate** | **Std Error** | **t Ratio** | **Prob>\|t\|** | **Term** | **Estimate** | **Std Error** | **t Ratio** | **Prob>\|t\|** |
| Intercept | 4.7781 | 11.80486 | 0.4 | 0.724 | Intercept | 3.927636 | 6.135821 | 0.64 | 0.587 |
| Genotype[SBetaThal°] | -0.784141 | 2.275601 | -0.34 | 0.763 | Genotype[SBetaThal°] | -0.540271 | 2.392125 | -0.23 | 0.842 |
| Genotype[SC] | 0.704262 | 2.387712 | 0.29 | 0.795 | Genotype[SC] | 0.493756 | 2.32398 | 0.21 | 0.851 |
| **WholeBrain_mean_ALFF** | 7.923592 | 233.4856 | 0.03 | 0.976 | **Csf_mean_ALFF** | 10.14013 | 48.7109 | 0.21 | 0.854 |
| **Term** | **Estimate** | **Std Error** | **t Ratio** | **Prob>\|t\|** | **Term** | **Estimate** | **Std Error** | **t Ratio** | **Prob>\|t\|** |
| Intercept | 5.17625 | 1.067029 | 4.85 | 0.016* | Intercept | -0.900942 | 11.96572 | -0.08 | 0.946 |
| Genotype[SBetaThal°] | -0.82125 | 1.629914 | -0.5 | 0.649 | Genotype[SBetaThal°] | 0.005317 | 2.479136 | 0 | 0.998 |
| Genotype[SC] | 0.74875 | 1.629914 | 0.46 | 0.677 | Genotype[SC] | 0.17103 | 2.192182 | 0.08 | 0.944 |
| **WholeBrain_mean_mALFF** | 0 | 0 | . | . | **Csf_mean_mALFF** | 2.507136 | 4.910322 | 0.51 | 0.660 |
| **Term** | **Estimate** | **Std Error** | **t Ratio** | **Prob>\|t\|** | **Term** | **Estimate** | **Std Error** | **t Ratio** | **Prob>\|t\|** |
| Intercept | 5.048669 | 1.254728 | 4.02 | 0.056 | Intercept | -5.753121 | 4.410875 | -1.3 | 0.322 |
| Genotype[SBetaThal°] | -1.006704 | 1.913021 | -0.53 | 0.651 | Genotype[SBetaThal°] | 2.024366 | 1.501433 | 1.35 | 0.31 |
| Genotype[SC] | 0.6375 | 1.890723 | 0.34 | 0.768 | Genotype[SC] | -0.435621 | 1.089502 | -0.4 | 0.727 |
| **WholeBrain_mean_zALFF** | -3.45E+08 | 6.77E+08 | -0.51 | 0.661 | Csf_mean_zALFF | 4.610787 | 1.840971 | 2.5 | 0.129 |
| **Grey Matter** | | | | | **White Matter** | | | | |
| **Term** | **Estimate** | **Std Error** | **t Ratio** | **Prob>\|t\|** | **Term** | **Estimate** | **Std Error** | **t Ratio** | **Prob>\|t\|** |
| Intercept | 4.840964 | 11.79055 | 0.41 | 0.721 | Intercept | 3.677897 | 13.48579 | 0.27 | 0.810 |
| Genotype[SBetaThal°] | -0.792116 | 2.240541 | -0.35 | 0.757 | Genotype[SBetaThal°] | -0.5726 | 2.986959 | -0.19 | 0.865 |
| Genotype[SC] | 0.712137 | 2.370787 | 0.3 | 0.792 | Genotype[SC] | 0.573398 | 2.535321 | 0.23 | 0.842 |
| **GreyMatter_mean_ALFF** | 6.859383 | 239.7291 | 0.03 | 0.979 | **WhiteMatter_mean_ALFF** | 40.97364 | 367.0546 | 0.11 | 0.921 |
| **Term** | **Estimate** | **Std Error** | **t Ratio** | **Prob>\|t\|** | **Term** | **Estimate** | **Std Error** | **t Ratio** | **Prob>\|t\|** |
| Intercept | 189.2814 | 645.5504 | 0.29 | 0.79 | Intercept | 2.53078 | 26.57527 | 0.1 | 0.932 |
| Genotype[SBetaThal°] | 0.411993 | 4.746414 | 0.09 | 0.938 | Genotype[SBetaThal°] | -0.612027 | 2.893445 | -0.21 | 0.852 |
| Genotype[SC] | 0.280914 | 2.553471 | 0.11 | 0.922 | Genotype[SC] | 0.732873 | 1.997652 | 0.37 | 0.748 |
| **GreyMatter_mean_mALFF** | -189.2117 | 663.4548 | -0.29 | 0.802 | **WhiteMatter_mean_mALFF** | 3.639599 | 36.51785 | 0.1 | 0.929 |
| **Term** | **Estimate** | **Std Error** | **t Ratio** | **Prob>\|t\|** | **Term** | **Estimate** | **Std Error** | **t Ratio** | **Prob>\|t\|** |
| Intercept | 4.749164 | 8.293154 | 0.57 | 0.624 | Intercept | 2.556499 | 8.949099 | 0.29 | 0.802 |
| Genotype[SBetaThal°] | -0.987241 | 3.756467 | -0.26 | 0.817 | Genotype[SBetaThal°] | -1.277843 | 2.490175 | -0.51 | 0.658 |
| Genotype[SC] | 0.831478 | 2.548746 | 0.33 | 0.775 | Genotype[SC] | 0.889674 | 2.011203 | 0.44 | 0.701 |
| **GreyMatter_mean_zALFF** | 17.05447 | 327.0319 | 0.05 | 0.963 | **WhiteMatter_mean_zALFF** | -5.807422 | 19.63452 | -0.3 | 0.795 |

* Indicates statistical significance *p* < 0.05.
